# Evaluation of grain yield stability of tritipyrum as a novel cereal in comparison with triticale lines and bread wheat varieties through univariate and multivariate parametric methods

**DOI:** 10.1371/journal.pone.0274588

**Published:** 2022-09-29

**Authors:** Sara Farokhzadeh, Hossein Shahsavand Hassani, Ghasem Mohammadi-Nejad, Zahra Zinati

**Affiliations:** 1 Department of Agronomy and Plant Breeding, Research and Technology Institute of Plant Production (RTIPP), College of Agriculture, Shahid Bahonar University of Kerman, Kerman, Iran; 2 Department of Crop Production and Plant Breeding, College of Agriculture, Shiraz University, Shiraz, Fars, Iran; 3 Department of Agroecology, College of Agriculture and Natural Resources of Darab, Shiraz University, Shiraz, Fars, Iran; Institute of Genetics and Developmental Biology Chinese Academy of Sciences, CHINA

## Abstract

Salinity is a major abiotic stress affecting cereal production. Thus, tritipyrum (x. *Tritipyrum*), a potential novel salt-tolerant cereal, was introduced as an appropriate alternative for cereal production. The purposes of this study were to evaluate agronomic traits, yield, and yield stability of eight primary tritipyrum lines, five promising triticale lines, and four bread wheat varieties and to screen a stable yielding line. The experiments were conducted in randomized complete block designs with three replicates in three locations during four growing seasons. Analysis of variance in each environment and Bartlett’s test for the variance homogeneity of experimental errors were made. Subsequently, separate experiments were analyzed as a combined experiment. The stability of grain yield was analyzed according to Eberhart and Russell’s regression method, environmental variance, Wrick’s ecovalance, Shokla’s stability variance, AMMI, and Tai methods. Genotype × environment interactions (GEI) and environments were significant for the agronomic traits. Stability analysis revealed that combined primary tritipyrum line (Ka/b)(Cr/b)-5 and triticale 4115, 4108, and M45 lines had good adaptability in all environments. The results of the AMMI3 model and pattern analysis showed that the new cereal, tritipyrum, had the most stable response in various environments. The tritipyrum line (Ka/b)(Cr/b)-5 had the best yield performance and general adaptability. Based on Tai’s method, the contribution of spike number to the stability of grain yield over different environments was higher than that of other yield components. Also, tritipyrum lines demonstrated higher stability compared with wheat and triticale. Totally, M45 triticale and tritipyrum (Ka/b)(Cr/b)-5 lines were the most stable genotypes with high grain yield. Complementary agronomic experiments may then release a new grain crop of triticale and a new pasture line of combined primary tritipyrum for grain and forage. Moreover, the combined tritipyrum line can be used in bread wheat breeding programs for producing salt-tolerant wheat cultivars.

## Introduction

Despite innovations in the green revolution such as developing genetically modified crops, world population growth continues to advance at a faster rate than food production. On the other hand, the worsening global climate increases stress on existing food production systems. So, to fill the expected gap in food production by 2050, the next green revolution is needed. The next green revolution uses conventional crossing and selection approaches, along with genetic modification. Interspecific hybrids or synthetic polyploids contribute most to crop performance under stress because wild species are often better adapted to stress conditions, or possess novel agronomic traits [1]. Triticale and tritipyrum are synthetic amphiploids which have been studied for their potential as alternative cereals adapted to abiotic stresses [2, 3]. Tritipyrum is made from the cross between durum wheat (2n = 4x = 28, AABB) and a wild grass of coastal salt named *Thinopyrum bessarabicum* (2n = 2x = 14, E^b^E^b^), to transfer salt tolerance genes and can potentially be used as an alternative to wheat in these areas [4, 5]. The incorporation of *Thinopyrum bessarabicum* E^b^ genome into wheat has contributed to salinity and drought tolerance and disease resistance [6]. *Thinopyrum bessarabicum* is recognized for a high tolerance of 350 mM of NaCl. However, 6x non-Iranian primary tritipyrum lines can withstand 250 mM NaCl [7]. *Thinopyrum bessarabicum* E^b^ genome, with a source of genetic material for adaption and tolerance to environmental stress, allows cultivating synthetic-derived wheat even in arid and semiarid regions. In a study, adaptation and agronomic traits of nine tritipyrum lines were evaluated in comparison with five triticale lines and four wheat cultivars. The overall results showed a large variation for all characters, implying a considerable potential for tritipyrum improvement as a new cereal compared with triticale lines and wheat cultivars [5]. The OPF03 primer could be used as a marker to identify the E^b^ genome in all the tritipyrum lines and materials with the E^b^ chromosome. In this regard, genomic DNA amplified with primer OPF03 showed the presence of a 1296-bp DNA fragment of the E^b^ genome in tritipyrum and *thinopyrum bessarabicum* but its absence in wheat breeding cultivars, Chinese spring wheat, and triticale promising lines [7]. From a molecular perspective, according to combined proteomic and transcriptomic analysis results of tritipyrum and the salinity-sensitive Chinese spring wheat, the high salt tolerance of tritipyrum could be pertinent to osmoregulation, enhanced respiration, reactive oxygen species scavenging, strengthened cell walls, phytohormone regulation, transient growth arrest, transcriptional regulation and error information processing [8].

Comparative assessment of physiological parameters in tritipyrum, wheat and triticale showed that tritipyrum species had the highest mean values for substomatal CO2 concentration (in tillering, early and late grain filling stages), net photosynthesis rate (in late grain filling), transpiration rate (in early grain filling), and stomatal conductance (in early and late grain filling stages) traits which accentuate the breeding potential of tritipyrum [9]. Several synthetic-derived lines, obtained by crossing tetraploid wheat and *Aegilops tauschii* Coss, showed higher photosynthetic rates than their recurrent parent. The maximum photosynthetic rate was negatively associated with leaf area and positively associated with stomatal and mesophyll conductance and leaf temperature depression. Photosynthesis is the primary physiological determinant of crop yield. Classical plant breeding and advances in agricultural approaches have yielded in higher-yielding plant varieties with efficiency enhancement at light-capture [10]. Accordingly, synthetic-derived wheat can also be a source of genetic diversity for important physiological traits such as enhanced photosynthetic rate [11]. Novel varieties with improved photosynthetic apparatus are more tolerant to environmental changes and efficient in the use of water and mineral nutrition resources [10].

Comprehensive reviews on triticale and tritipyrum response and adaptation show that they can tolerate some abiotic stresses such as salinity better than small grain cereals such as barley, rye and oats [12–14]. In a study conducted by Shahriari et al. [15], mitotic instability of seven primary tritipyrum was evaluated in comparison to wheat and triticale. Cytological investigations showed that the incidence of aneuploidy in tritipyrum was significantly higher than wheat and triticale. Moreover, aneuploidy had a significant negative correlation with 1000-grains weight, grain yield and fertility in tritipyrum. Mitotic instability was significantly higher in light grains than heavy grains. Although the chromosomal instability has made primary tritipyrum not yet considered as a salt-tolerant commercial crop, they are prone to be another successful man-made cereal [15]. In order to remove undesirable traits in the non-Iranian primary tritipyrum lines (NIPTLs), the crossing of NIPTLs with Iranian bread wheat cultivars was made and led to new recombinant Iranian secondary tritipyrum lines (ISTLs) [14]. Roudbari et al. [14] evaluated 13 NIPTLs and 92 ISTLs, 6 bread wheat cultivars and 1 triticale line using an alpha lattice design with two replications under normal and salinity stress (12 dS.m^-1^) conditions during two crop years. Their results indicated which ranking of lines, based on breeding values with the best linear unbiased prediction (BLUP), is a good way to select salt-resistant lines with high yield potential. Also, they reported the (Cr/b)×(Ka/b) line of NIPTLs and the lines obtained from Niknejad × (Ka/b)(Cr/b) and Omid × (Ka/b)(Cr/b) crosses, had the highest average breeding value, that will be appropriate for breeding programs with high yield potential in saline soils and waters. Pourfereidouni et al. [16] reported that ISTLs are superior to NIPTLs in terms of morphological traits. Self-crossing or backcrossing of NIPTLs with bread wheat cultivars has led to stability in progeny and a decrease in aneuploidy rates over several generations. The results of Khalifeie and Mohammadi-Nejad [17] showed a high tolerance of NIPTLs to salinity compared to wheat and triticale.

According to other research and based on their seed maturity traits, reproductive, and vegetative, tritipyrum lines had greater salt tolerance than salt-tolerant wheat cultivars [18]. Besides, according to field trials, tritipyrum lines produced a higher grain yield as well as higher grain protein content and displayed better performance than wheat cultivars in salinity condition. However, a few studies have been done on the cultivability and yield stability of these new crops worldwide [19]. Genotype-by-environment interaction (GEI) is often a great challenge for breeders since it makes the selection of stable or superior genotypes more difficult [20]. Accurate determination of yield stability of genotypes and quality traits is often difficult owing to the GEI [21]. Previous studies have analyzed GEI to improve crop breeding and selection of high-yielding and stable varieties [22–25]. Generally, the interaction between the genotype and environment had made it challenging to find superior and more stable genotypes [26–28]. To achieve stable yield production, the development of genotypes with a consistent high yield in various environments (E) along with good grain quality is inevitable [29, 30]. The best way to overcome this problem is to assess genotypes across a diverse set of environments over several years under different conditions [26, 27, 31].

The GEI provides valuable information concerning plant yield in different environments and plays an important role in the evaluation of the functional stability of breeding material [26]. Stable genotypes show similar responses in various environments [32]. But, GEI can affect the yield of genotypes and lead to yield difference in different environments [33]. Many approaches have been utilized to determine and unravel the causes of interactions, although strategies are different in the final decisions for selecting genotypes [34, 35]. Researchers have proposed various methods for stability analysis [36–38]. There is often a linear or near-linear relationship between the appearance of traits in different genotypes and the environmental effect, which is usually measured by different criteria. Therefore, Yates and Cochran [39] proposed the regression method to evaluate the response of genotypes to different environmental conditions. The regression coefficient index was first used by Finlay and Wilkinson [40] and then by Eberhart and Russel [41] to show the adaptation of genotypes to environmental changes. Finlay and Wilkinson [40] stated that the regression coefficient (b_i_) was a measure of genotype adaptability and stability. In addition to two recent criteria, Eberhart and Russel [33] used deviations from the regression line (S^2^_di_) as another criterion to identify stable varieties. In their opinion, ideal genotypes must be with a high yield, a regression coefficient equal to one (b_i_ = 1), and a deviation from regression as small as possible (S^2^_di_ = 0). Pinthus [42] suggested that the detection coefficient (R^2^) be used instead of the square mean of deviation of the regression line because R^2^ is highly dependent on S^2^_di_. The environmental variance (S_i_^2^) is another stability index. According to this index, the stable genotype has the smallest environmental variance. The use of the environmental variance index is more effective in geographical range with low diversity [43]. Francis and Kannenberg [44] introduced the coefficient of variation (CV) related to each genotype as a stability parameter and recognized genotypes with more yields than the mean and the coefficient of variation of less than mean as stable genotypes. Lin et al. [43] also stated that if the researcher is interested in determining stability in a certain range of environmental conditions, the stability index of the coefficient of variation is a useful criterion. Wricke [45] introduced another stability index (W^2^_i_) which was actually the sum of squares of GE interactions for each genotype. Shukla [46] proposed the stability variance index (σ^2^_i_) for each genotype. According to the two mentioned methods, genotypes are considered stable and the value of each of the two recent indexes is minimal in them [47]. Akcura et al. [34] used parameters of the b_i_, S^2^_di_, R^2^_i_, CV_i_, Shukla stability variance (σ^2^_i_), Wrick ecovalance (W^2^_i_), environmental variance (S^2^_i_), and the stability parameters of the Tai method for stability analysis of durum wheat genotypes, and finally, they introduced five stable genotypes.

Although several stability measures have been developed to assess the stability and adaptability of genotypes, multivariate statistical methods are more efficient than conventional univariate techniques, due to the description of GEI in multidimensional models [48]. In recent years, the AMMI multivariate model has been used as a powerful analytical tool to study GEI in large matrix data structures [49]. The AMMI model combines ANOVA and principal component analysis (PCA) where the sources of variability in the genotype by environmental interaction are partitioned by PCA. The explanation of results obtained from the AMMI model is accomplished with a biplot that relates genotypic means to the first or some of the principal interaction components [49]. The AMMI model has been presented to be an efficient method because it justifies a large portion of the GE sum of squares and uniquely separates main and interaction effects, as required for most agricultural research goals [50]. Tarakanovas and Ruzgas [51] introduced the AMMI model as an effective method to study the GEI and stated that the results obtained by its biplot can determine the suitable genotypes for planting in different environments or specific environmental conditions. Mohammadi et al. [52] reported the significant interaction of the four first principal components in pattern analysis of durum wheat, in which 65% of the sum of squares of the interaction was expressed by two principal components.

Thomas et al. [53] offered a method to study GEI and stated that the growth and development of a crop is a complex developmental system and the grain yield is the result of the cumulative effects of its constituent components. Therefore, the identification of these components and their relationship with the yield can be effective in selecting high-yield and stable genotypes. Each component of this system is also affected by plant genotype, environmental conditions, and their interaction, and environmental factors have a different effect on them. Accordingly, Tai [54] used path coefficient analysis to GEI analyze and determine the contribution of genotypic and environmental components in its formation. A highly significant difference was reported among the 19 wheat lines for grain yield and also, genotype × year interaction in the evaluation of grain yield using the path analysis method by Soughi et al. [55]. They reported that the direct effect of 1000-grain weight was negligible, but the direct effect of the grain number per spike was high for selecting superior lines [55].

Although the tolerance to salinity and drought of tritipyrum has been well documented, a few studies have been done on the cultivability and yield stability of these new crops worldwide. So in this study, we tried to evaluate the grain yield stability of tritipyrum in three locations with arid and semi-arid climates, in comparison with triticale lines and bread wheat varieties. The objects of the current study were to: a) investigate the GEI of new cereal, non-Iranian primary and combined primary tritipyrum lines, promising triticale lines and bread wheat varieties under various environmental conditions for grain yield (t.ha^-1^) and its components using univariate and multivariate parametric methods, b) Determine the contribution of each environmental factor in creating the GEI using Tai’s path coefficient analysis method, c) Comparison of yield potential and adaptability of new cereal, primary tritipyrum lines with Iranian bread wheat varieties and promising triticale lines, d) Identify the genotypes with stable yield, and (e) study the correlation between the stability parameters.

## Materials and methods

**Fig 1:**
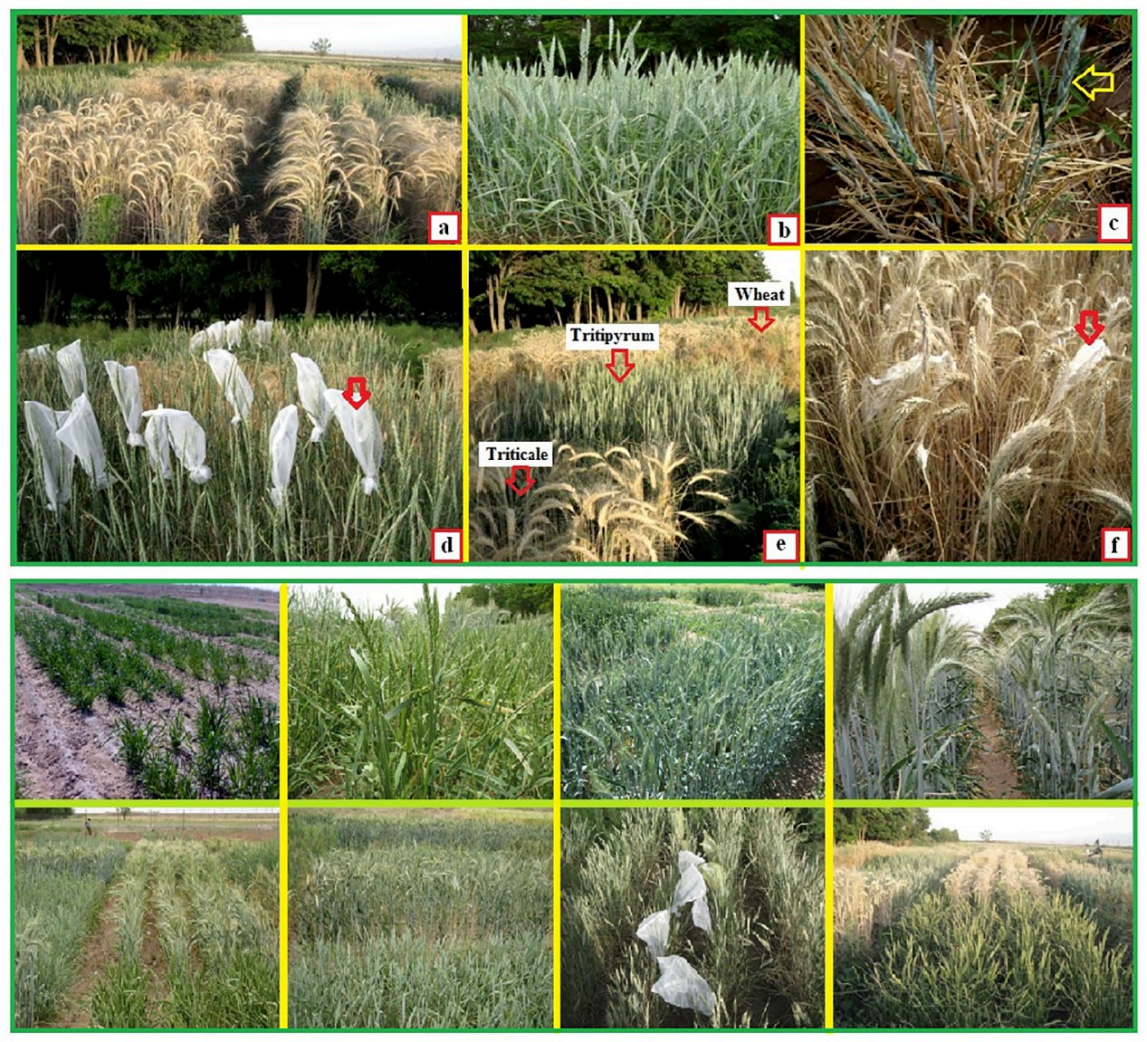
(a) Experimental design of three hexaploid amphiploids including eight non-Iranian primary and combined primary tritipyrum lines, five promising triticale lines and four bread wheat varieties in the field of agricultural research station of Shahid Bahonar university, Iran, (b) morphology of the silver color index of the primary and combined primary tritipyrum lines, (c) the characteristic of continuous tillering and flowering of the primary and combined primary tritipyrum lines, (d) prevention of seed falling before harvest of the primary and combined primary tritipyrum lines, (e) delay in the maturity of primary and combined primary tritipyrum lines in comparison with triticale lines and bread wheat varieties, (f) determining spike samples with plastic cover for measuring morphological and agronomic traits, and the rest of the figures are related to the studied experimental environments.

Three hexaploid amphiploids, including eight non-Iranian primary and combined primary tritipyrum (2n = 6x = 42, AABBE^b^E^b^) lines, five promising triticale (2n = 6x = 42, AABBRR) lines and four Iranian bread wheat (2n = 6x = 42, AABBDD) varieties were evaluated in this study (Fig 1 and S1 Table in S1 File).

In each trial (environment), All genotypes were planted in a randomized complete block (RCBD) design with three replications in three locations of Iran (Kerman, Sirjan, Neyriz), during four growing seasons {e_1_: Kerman (normal) and fourth crop year; e_2_: Kerman (normal) and second crop year; e_3_: Kerman (normal) and third crop year; e_4_: Sirjan (normal) and fourth crop year; e_5_: Neyriz (normal) and first crop year; e_6_: Kerman (normal) and first crop year; and e_7_: Sirjan (salinity, Ec = 15 dS.m^-1^) and fourth crop year} (Fig 1 and S2 Table in S1 File).

The required amount of seed was calculated based on the thousand-grain weight and grain number per square meter. The plots consisted of four 3 m long rows with 0.5 m spacing between the rows (plot size: 6 m^2^). The 30 seeds were planted manually with 10 cm space in one row on each ridge (120 seeds in each plot). To minimize other grain yield-reducing factors, carboxin thiram fungicide was used to control diseases. Also, weed control was performed in all stages of crop growth in all trials. All the trials received nitrogen (N, kg.ha^-1^) fertilizer in three stages: before planting, one month after planting, and before flowering. At the sowing stages, phosphorus (P, kg.ha^-1^) fertilizer was also applied. The middle rows of each plot were used for data collection, to eliminate the effects of neighboring genotypes for water, light, and the essential resources for canopy growth. Data for agronomic traits were recorded as follows:

Days to heading: was calculated when 50% of spikes emerged from the flag leaf sheath.
The total tiller number per plant and fertile spike number per plant: were recorded from 10 randomly selected plants grown in the center rows of each plot.
Plant height (cm): was measured as the distance from the ground level to the tip of the spike (excluding the awns) of ten plants per plot.
Spike length (cm): was measured from the base of the rachis to the tip of the terminal spikelet excluding the awns in 10 leading spikes after harvest.
Spikelet number per spike: was determined by counting the number of fertile and sterile spikelets of 10 leading spikes after harvest.
Grain number per spike: was recorded from 10 randomly selected spikes grown in the center rows of each plot. Grains from this sample of 10 spikes were threshed and counted.
1000-grain weight (g): was measured by weighing random samples of harvested grains.
Grain yield (t.ha^-1^): was estimated by weighting grains of harvested plants in each plot (g.m^-2^), when the grains were dry at about 4%–5% humidity, then converted into t.ha^-1^
Harvest index (HI, %): was calculated by the following equation:
$$\text{HI}=\left(\text{grain}\;\text{yield}/\text{biological}\;\text{yield}\right)\times 100.$$

### Analysis of variance

Analysis of variance in each environment was done, separately and also, Bartlett’s test for the variance homogeneity of experimental errors was examined. Subsequently, separate experiments were analyzed as a combined experiment. In this experiment, genotypes and environments were considered as fixed and random effects, respectively. Mean comparisons were done using Duncan’s multiple range tests at the 0.05% probability level.

### Estimation of stability parameters

The univariate and multivariate parametric stability analyses were performed to evaluate genotypes grain yield throughout multiple environments and predict stable genotypes.

#### Univariate stability analysis

*Eberhart and Russell’s regression method*. The method of Eberhart and Russell [41] was used in this study to characterize genotypic stability. The linear regression:

$$Y_{ij}=m+\beta_{i}I_{j}+S_{ij}$$

Where Y_ij_ is the mean of the i^th^ genotype in the j^th^ environment, m = is the mean of all genotypes in all environments, β_i_ is the regression coefficient of the i^th^ genotype on the environmental index which measures the response of genotypes to a different environment, I_j_ is the environmental index which is defined as the mean deviation of all genotypes in the j^th^ environment from the overall mean, S_ij_ is the deviation from regression of the i^th^ genotype at the j^th^ environment.

It is worth mentioning that: $Ij=\frac{\sum_{j}y_{ij}}{p}-\frac{\sum_{i}\sum_{j}y_{ij}}{pq}$, Σ_*i*_ *I*_*J*_ = *o*, p and q are the number of genotypes and experimental environments, respectively.

Two stability parameters were calculated: (a) the regression coefficient, which is the regression of the performance of each genotype in different environments calculating environmental means over all the genotypes. This is estimated, according to Sing and Chaudhary [1979] as follows

$$b_{i}=\frac{\sum_{j}y_{ij}\;I_{j}}{\sum_{j}I_{j}^{2}},$$

Where Σ_*j*_ *y*_*ij*_ *I*_*j*_ is the sum of products and $\sum_{j}I_{j}^{2}$ is the sum of squares.

(b) Mean square deviations (s^2^d_i_) from linear regression

S2di=∑iδij2(q−2)−S2er;∑iδij2=(∑jYij2−y¯i2.p)−∑jYijIj∑jIj22

Where s^2^e is the estimate of pooled error and r is the number of replications in each experiment.

The linear regression coefficient (b_i_) of the relationship between the yield for the genotype in each environment and the yield for the mean environment is a measure of the linear responses to environmental change. The mean square of deviation from the regression (s^2^d_i_) measures the consistency of this response: in other words, it is a measure of heterogeneity.

*Environmental variance (S*^*2*^_*i*_*)*. The environmental variance of genotypes (S^2^_i_) is calculated by Roemer [56] to determine the stability of a genotype using the formula:

$$S_{i}^{2}=\sum_{j=1}^{q}{\left(y_{ij}-{\bar{y}}_{i}.\right)}^{2}/q-1$$

Where y_ij_ is the mean value of the yield for the i^th^ genotype in the j^th^ environment, ${\bar{y}}_{i}.$ is the mean of the yield of i^th^ genotype in all environments, and q is the environments number. The most stable genotypes have the lowest environmental variance. In fact, S^2^_i_ is an unbiased estimation of genotype variation.

*Coefficient of variation (CV)*. Francis and Kannenberg [44] suggested the use of CVi=Siy¯i.×100 as a combination of mean yield and standard deviation to measure of genotype stability. Where S_i_ is the standard deviation of the yield for the i^th^ genotype, ${\bar{y}}_{i}.$ is the mean of the yield of the i^th^ genotype in all environments. Genotypes with CV_i_ below overall coefficient of variation and yield above the overall mean yield are considered more stable than the others.

*Wrick ecovalance (W*^*2*^_*i*_*)*. Wricke [45] proposed the idea of ecovalence parameter to calculate the share of each genotype to the sum of squares of GEI by using the equation:

$$W_{i}^{2}=\sum_{j=1}^{q}{\left(y_{ij}-{\bar{y}}_{i}.-{\bar{y}}_{j}+\bar{y}..\right)}^{2}$$

Here, yij represents the mean of i^th^ genotype in the j^th^ environment, ${\bar{y}}_{i}.$ is the mean of the yield of i^th^ genotype in all environments, ${\bar{y}}_{j}$ is the mean yield of the genotypes in the j^th^ environment and $\bar{y}..$ is the grand mean. The sum of ecovalence values for all genotypes is equal to the sum of squares of the GEI. In other words: $\sum W_{i}^{2}=SSGE$; thus, any genotype with W^2^_i_ = 0 is stable. Unstable genotypes have high ecovalence.

*Shukla stability variance (σ*^*2*^_*i*_*)*. Shukla [46] introduced Shukla’s stability variance of genotypes across different environments based on the equation:

$$\delta_{i}^{2}=\frac{P}{\left(P-2)(q-1\right)}W_{i}^{2}-\frac{SSGE}{\left(P-1)(P-2)(q-1\right)}$$

Here, p and q represent the genotypes and environments number, while W^2^_i_ is Wricke’s ecovalence of the i^th^ genotype. The sum of squares of the GEI is obtained as follows:

$$SSGE=\sum_{}W_{i}^{2}-\;\sum_{i}\sum_{j}{\left(y_{ij}-{\bar{y}}_{i.}-{\bar{y}}_{.j}+\bar{y}..\right)}^{2}$$

Stability variance is a linear combination of ecovalence. Therefore, these have the same value in terms of genotype ranking.

#### Multivariate stability analysis

*AMMI method*. In the AMMI model, the parameters of GEI, Eigen value, and principal components values were computed for genotypes and environments. These values were used to evaluate the stability of genotypes and environments in the biplot and also to calculate the stability parameters of AMMI model. It was also used to identify genotypes with broad or specific adaptation to target environments for grain yield [57]. The AMMI model is expressed as:

$$Y_{ijK}=\mu +g_{i}+e_{j}+\sum_{n=1}^{N}\delta_{n}\zeta_{in}\eta_{jn}+\theta_{ij}+\varepsilon_{ijk}$$

where Y_ij_ is the yield of the i^th^ genotype in the j^th^ environment in the k replication, μ is the overall mean, g_i_ is the main effect of the i^th^ genotype and e_j_ is the main effect of the j^th^ environment (g_i_ and e_j_ are the genotype and environment deviations from the grand mean, respectively), σ_n_ is the square root of the eigenvalue of the PCA axis n (λ^0.5^), ζ_in_, η_jn_ are the principal components scores for principal component (PCA) n axis of the i^th^ genotype and j^th^ environment, respectively, *θ*_*ij*_ is the residual (noise) amount of the AMMI model, *ε*_*ijk*_ is the model error and n is the number of interaction principal components (IPC) in the AMMI model, which is equal to [*n*≤min (*g*−1), (*e*−1)].

The SIPC (Sum of IPC scores) parameter was also computed by Sneller et al. [58] as:

$$SIPC=\sum_{n=1}^{N}\left|\lambda^{0.5}\zeta_{in}\right|,$$

in this equation N = 1 for SIPC1; for SIPCF, N was the number of IPC that were retained in the AMMI model.

The eigenvalue (EV) stability parameter of AMMI was computed by Zobel [59] according to the equation:

$$EV=\frac{\sum_{n=1}^{N}\zeta_{in}^{2}}{N},$$

in this formula, N is the number of IPC that were retained in the AMMI procedure via different F-tests.

The biplot diagram was used in order to investigate the stability of genotypes, to evaluate changes in environments, and also to relate stable genotypes to different environments. In addition, in order to pattern analysis and the simultaneous use of classification and vectorization methods and more accurate examination of the stability of genotypes, the results of cluster analysis based on the values of the main components of genotypes and environments are also shown on biplot diagrams.

**Fig 2:**
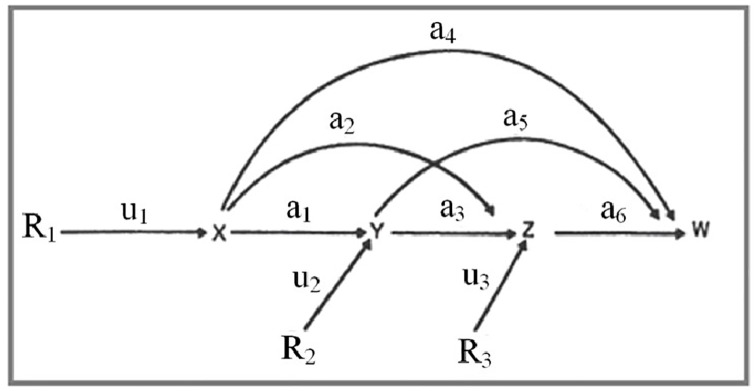
Causation diagram showing the path relationships between grain yield and yield components and environmental components on yield.

*Path analysis of GEI (Tai method)*. The contribution of each trait in the GEI was determined using the Tai model and stability analysis based on the path coefficient analysis [54]. In this research, the X (spike number), Y (grain number per spike) and Z (1000-grain weight) were assumed to be sequential traits justifying grain yield productivity (W). Path relationships between grain yield and yield components and environmental components on yield are shown in Fig 2.

R_1_, R_2_ and R_3_ are environmental components influencing X, Y and Z, respectively. u_1_, u_2_ and u_3_ are the path coefficients from R_1_ to X, R_2_ to Y and R_3_ to Z, respectively. a_1_-a_6_ are path coefficients of X with Y, X with Z, Y with Z, X with W, Y with W, and Z with W, respectively.

The yield of the i^th^ genotype in the j^th^ environment can be expressed as:

$$W_{ij}=\mu_{w}{}_{i}+V_{1i}r_{1j}+V_{2i}r_{2j}+V_{3i}r_{3j}+e_{ij}$$

The observed yield (W_ij_) is composed of a mean genotypic effect (μ_wi_), three multiplicative terms representing the GEI effects formed by three genotypic components (V_1i_, V_2i_ and V_3i_), three environmental components (r_1j_, r_2j_ and r_3j_) and an error deviate (e_ij_). The three genotypic components indicate the efficiency of the genotype in using environmental components during the stages of plant development in the formation of final yield. Each of the environmental components indicates the relative importance of that environmental factor on the yield-related components, which is constant in each environment. The higher the absolute value of r for a trait, it means that the trait is more influenced by the environment and has less stability. In fact, this method is used to determine which genotype in which stage of development was the most sensitive to environmental factors [54]. In order to investigate GEI using path analysis, correlation coefficients between the yield and its components for different genotypes were determined, separately, and the direct effects of traits on yield, the effects of environmental factors on the yield and its components were calculated for each genotype. Finally, stable genotypic components were determined for the yield components of each genotype and the environmental components affecting them during the growth stages [54].

### Correlation of stability parameters

Spearman‘s rank correlation coefficient was calculated between mean yield and stability parameters to compare the described methodologies [34].

Stability analyses were performed using SAS (Statistical Analysis System, version 9.2), MATLAB (MATrix LABoratory, R2020b, version 9.9), SPSS (Statistical Package for the Social Sciences, version 24), S116, R and RStudio (version 4.0.3) and EXCEL (2013) software.

## Results and discussion

### Effect of the genotype (G), environment (E) and their interaction (G×E)

The non-significant chi-square value in Bartlett’s test indicated the uniformity of error variance in seven environments for grain yield and agronomic traits (S3 Table in S1 File). The results of the combined analysis of variance showed significant differences between environments and considerable genotypic variation for grain yield (S4 Table in S1 File). Grain yield in the fourth and seventh environments (Sirjan) had a significant decrease compared to the third environment (Kerman). Therefore, in different planting environments, the presence of factors such as the heat requirement of plants, wind, light, moisture, nutrients, etc. is effective in increasing or decreasing grain yield (S1 Fig). There were highly significant differences between the means of triticale lines, primary tritipyrum lines, and wheat varieties for grain yield. Combined analysis of variance (S4 Table in S1 File) revealed significant GEI for grain yield. The significant GEI is reflected in the differential response of genotypes in diverse environments. This displayed that GEI was highly significant and had a considerable effect on genotypic performance in various environments. So, it was feasible to proceed and compute stability parameters.

In e_1_, the highest yield was related to Omid wheat, 4116, 4108, 4103 and (Ka/b)×(Cr/b)-5 lines, respectively. In e_2_, Omid and Kavir wheats, 4108 and M45 lines had the highest yield, respectively. While, La/b line had the lowest value in e_1_, e_2_, and e_6_. In e_3_, The La(4B/4D)/b, (Ma/b)×(Cr/b)-4, La/b, (Ka/b)×(Cr/b)-5 tritipyrum lines, respectively, had more yield compared with other genotypes. The highest and lowest yield was observed in the (4108, M45 and Ka/b) and (Ma/b)×(Cr/b)-4 genotypes, respectively, in the e_4_. The [4115, Baharebaft, 4108, 4116 and (Ka/b)×(Cr/b)-5] genotypes and (Ka/b)×(Cr/b)-6 line had the highest and lowest yield, respectively, in the e_5_. In e_6_, the highest grain yield was observed for (Kavir, Baharebaft and Alvand) wheats and (4115 and M45) triticale lines, respectively. The M45, 4108 and 4115 triticale lines and La(4B/4D)/b, (Ma/b)×(Cr/b)-4 tritipyrum lines showed the highest yield, respectively (S5 Table in S1 File). In general, La (4B,4D)/b, (Ma/b)×(Cr/b)-4 primary tritipyrum lines had the highest yield (11.5 and 11.36 t.ha^-1^), respectively in the third environment in Kerman region (e_3_), and (Ma/b)(Cr/b)-4 tritipyrum lines had the lowest yield (1.03 t.ha^-1^) in the fourth environment (e_4_) in the Sirjan region (S5 Table in S1 File). For grain yield, GE was accounted for 61.48% of the total sum of squares and was higher than the genotype and environment effects, which propose the possible existence of different environmental groups (S4 Table in S1 File). The large ratio of GE interaction in this study makes more differences in the genetic systems which control the physiological activities, conferring yield stability in various environments. Many other researchers also found a high level of GEI in their experiments [60–64]. The results of Mohammadi et al. [65] suggested that the GEI was related to the interaction of heading date, rainfall, freezing days, plant height, and air temperature.

### Eberhart and Russell’s regression method

The assessment of promising genotypes across diverse environments is an essential final stage in most applied plant breeding programs. As quantitative inherited attributes, grain yield may perform well in specific environments and vice versa in some others, leading to a meaningful GEI which can seriously restrict gains of selecting superior genotypes. Realization of the interaction of those agents and how they impact grain yield is important for maintaining high yields [66]. In this study, pooled analysis of variance of grain yield for 17 genotypes and varieties of three amphiploids using Eberhart and Russell^’^s regression method revealed highly significant differences among environments, GEI and combined deviations (S6 Table in S1 File). Significant combined deviations showed that deviation from the linear regression was significant for genotypes and varieties. So genotypes and varieties have unpredictable responses to environmental changes. Non-significant linear GEI showed that the response of different genotypes was similar to different environmental conditions.

The sum of squares of linear GE was significant only for La/b, (Ma/b)(Cr/b)-4, (Ka/b)(Cr/b)-3 and (Ka/b)(Cr/b)-5 tritipyrum lines, and 4103 triticale line and was non-significant for other genotypes. Thus, a linear relationship explains the yield changes of these genotypes in different environments (S7 Table in S1 File). Non-significant linear GEI is an indication of no significant difference between genotypes in terms of the slope of the regression line. In other words, the response of different genotypes is similar to different environmental conditions. Based on the result of stability analysis of the regression model of Finlay and Wilkinson [32], the lowest and highest linear regression coefficient belonged to Omid (b_1_ = 0.23) and (Ma/b)(Cr/b)-4 tritipyrum (b_2_ = 1.76), respectively (S7 Table in S1 File). Despite the high variation, the linear regression coefficients had no significant difference with one (b = 1). Among the triticale genotypes, 4103, 4115, and M45 lines had a regression coefficient close to one (b = 1) and only 4115 and M45 lines showed good general adaptability with higher yields than the mean, according to the stability graph of genotypes and varieties based on linear regression coefficient (S2 Fig). On the other hand, the M45 line with lower regression deviation variance and a high coefficient of determination should have better general adaptability than 4115 line. The Ka/b primary tritipyrum, (Ka/b)(Cr/b)-6 and (St/b)(Cr/b)-4 combined primary lines, 4108, 4116 triticale lines and Omid and Alvand wheat varieties had specific adaptation to unfavorable environments, while La/b, La (4B,4D)/b, (Ma/b)(Cr/b)-4, (Ka/b)(Cr/b)-3, (Ka/b)(Cr/b)-5 primary tritipyrum lines and Bahare baftand and Kavir wheat varieties showed specific adaptation to favorable environments (S2 Fig). To investigate individual deviation from the regression, F-test (S7 Table in S1 File) indicated that the mean squares of deviation from the regression are very significant for all genotypes, so the use of regression method alone is not sufficient in justification of GEI analysis, and this is one of the complications of regression method in the stability analysis [41].

**Fig 3:**
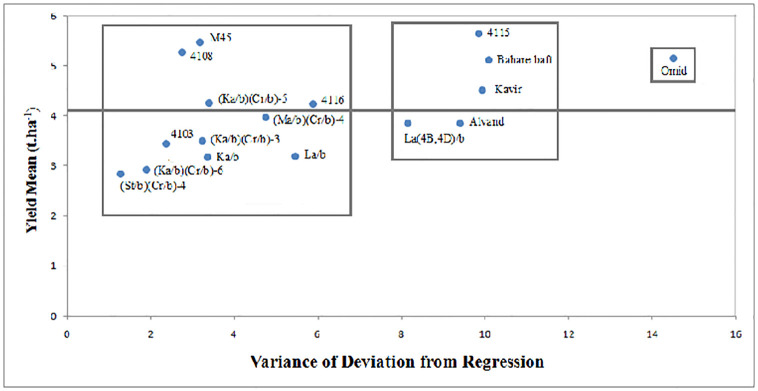
Biplot graph for three amphiploids of non-Iranian primary and combined primary tritipyrum lines, promising triticale lines and bread wheat varieties based on the mean of grain yield and the values of deviation from linear regression, the second parameter of Eberhart and Russell. Interconnected lines show grouping obtained from cluster analysis based on the variance of deviation from regression, and continuous horizontal lines pass through the mean grain yield.

The S^2^_di_ becomes a main statistic in estimating stability if the regression coefficients do not differ significantly [67]. In this study, based on the variance of deviation from the regression (S^2^_d_), genotypes were divided into three groups in cluster analysis using the ward method (Fig 3). The first group included Omid wheat variety as unstable with the highest deviation from regression. The second group with the intermediate s^2^d included La (4B,4D)/b primary tritipyrum line, 4115 triticale line and Alvand, Kavir and Bahare baft wheat varieties. The third group with low s^2^_d_ included Ka/b, La/b primary tritipyrum lines and (Ma/b)(Cr/b)-4, (Ka/b)(Cr/b)-3, (Ka/b)(Cr/b)-5, (Ka/b)(Cr/b)-6, (St/b)(Cr/b)-4 primary tritipyrum lines and 4103, 4108, 4116 and M45 triticale lines identified as stable lines. Also, 4108, 4116 and M45 promising lines and (Ka/b)(Cr/b)-5 primary line had higher yield than the mean, so they were considered as the lines with desired general stability (Fig 3).

The lowest and highest linear coefficient of determination related to the Omid wheat variety (R^2^ = 1.1%) and (Ka/b)(Cr/b)-5 tritipyrum line (R^2^ = 69.9%), respectively (S7 Table in S1 File). Low values of R² illustrate high scattering of the data and hence low reliability of the type of environmental response defined by the regression model [68]. Results of cluster analysis divided cultivars and lines into three groups based on R^2^ (S7 Table in S1 File). The first group included La/b primary tritipyrum line and three primary lines of (Ma/b)(Cr/b)-4, (Ka/b)(Cr/b)-3, (Ka/b)(Cr/b)-5 and 4103 triticale line with the highest R^2^. In the second group, two primary tritipyrum lines {Ka/b, La (4B,4D)/b} and two combined primary tritipyrum lines {(Ka/b)(Cr/b)-6, (St/b)(Cr/b)-4}, two promising triticale lines (4115, M45) and Bahare baft and Kavir wheat varieties were placed. Also, 4108, 4116 triticale lines, Omid and Alvand wheat varieties were placed in the third group with the lowest R^2^. Consequently, according to the mean yield and both stability parameters of Eberhart and Russell, M45 and 4115 lines have desired general stability. The GEI sum of squares explained only 11.81% of the total interaction of the sum of squares (S6 Table in S1 File). But researchers’ recommendations, including the Hayward et al. [69] suggest that this should be explained at least 50% of the total sum of squares by GEI for regression analysis to be useful. The efficiency of linear regression models is questionable when the heterogeneity of the slopes does not reach significance and illustrates a little part of the GEI [67]. Therefore, the use of this method alone is not enough for stability analysis, and it is necessary to use other stability statistics such as coefficient of variation and environmental variance to determine stable genotypes.

### Environmental variance, coefficient of variation, Wrick ecovalance and Shukla stability variance

The results of this study showed that (St/b)(Cr/b)-4 tritipyrum lines with the lowest environmental variance (S_i_^2^ = 1.51) and Bahare baft wheat with the highest environmental variance (S^2^_i_ = 14.23) are the most stable and unstable genotypes, respectively (Table 1). The (Ka/b)(Cr/b)-6 and 4108 lines were placed in the subsequent ranks of stability (Table 1). The environmental variance parameter is indicative of the biological concept of stability and the first group component of Lin and Binn’s stability [70]. According to the coefficient of variation (CV), 4108 triticale line and La/b primary tritipyrum line are the most stable and unstable genotypes, respectively (Table 1). Soughi et al. [71] introduced five stable genotypes using CV and S^2^_i_ parameters in their study on the grain yield stability of bread wheat lines in the northern warm and humid climate of Iran.

**Table 1:** Environmental variance, coefficient of variation, Wrick ecovalance and Shukla stability variance parameters for non-Iranian primary and combined primary tritipyrum lines, promising triticale lines and bread wheat varieties in the different environments.

Genotypes	Environmental variance (S^2^_i_)	Coefficient of variation (CV)	Wrick ecovalance (W^2^_i_)	Shukla stability variance (σ^2^_i_)
Ka/b	3.83	61.59	18.48	3.10
La/b	11.53	99.9	34.49	6.12
La(4B,4D)/b	11.97	89.93	43.96	7.91
(Ma/b)(Cr/b)-4	11.38	84.67	32.06	5.66
(Ka/b)(Cr/b)-3	6.95	75.65	17.80	2.97
(Ka/b)(Cr/b)-5	9.42	72.08	23.25	4.02
(Ka/b)(Cr/b)-6	2.44	53.45	11.77	1.83
(St/b)(Cr/b)-4	1.51	42.91	10.45	1.58
Triticale 4103	4.85	64.02	11.92	1.86
Triticale 4108	2.44	29.66	21.44	3.66
Triticale 4115	10.38	57.02	49.23	8.91
Triticale 4116	5.23	53.87	34.98	6.22
Triticale M45	4.52	38.84	16.10	2.65
Omid	12.21	68.51	80.73	14.86
Alvand	8.59	76.00	49.73	9.02
Baharebaft	14.23	73.64	55.17	10.03
Kavir	3.03	79.80	52.06	9.44

In the above-described situation, most of the genotypes were stable based on environmental variance; they were also detected as the stable genotypes in terms of coefficient of variation. That this is indicative of the similarity between two indices in the determination of the stable genotypes. Although the parameters of the environmental variance and coefficient of variation are heritability and can be a suitable criterion for selecting varieties, these methods can’t always be achieved to the most stable and high-yielding varieties. Thus, the use of other methods is essential alongside these methods.

**Fig 4:**
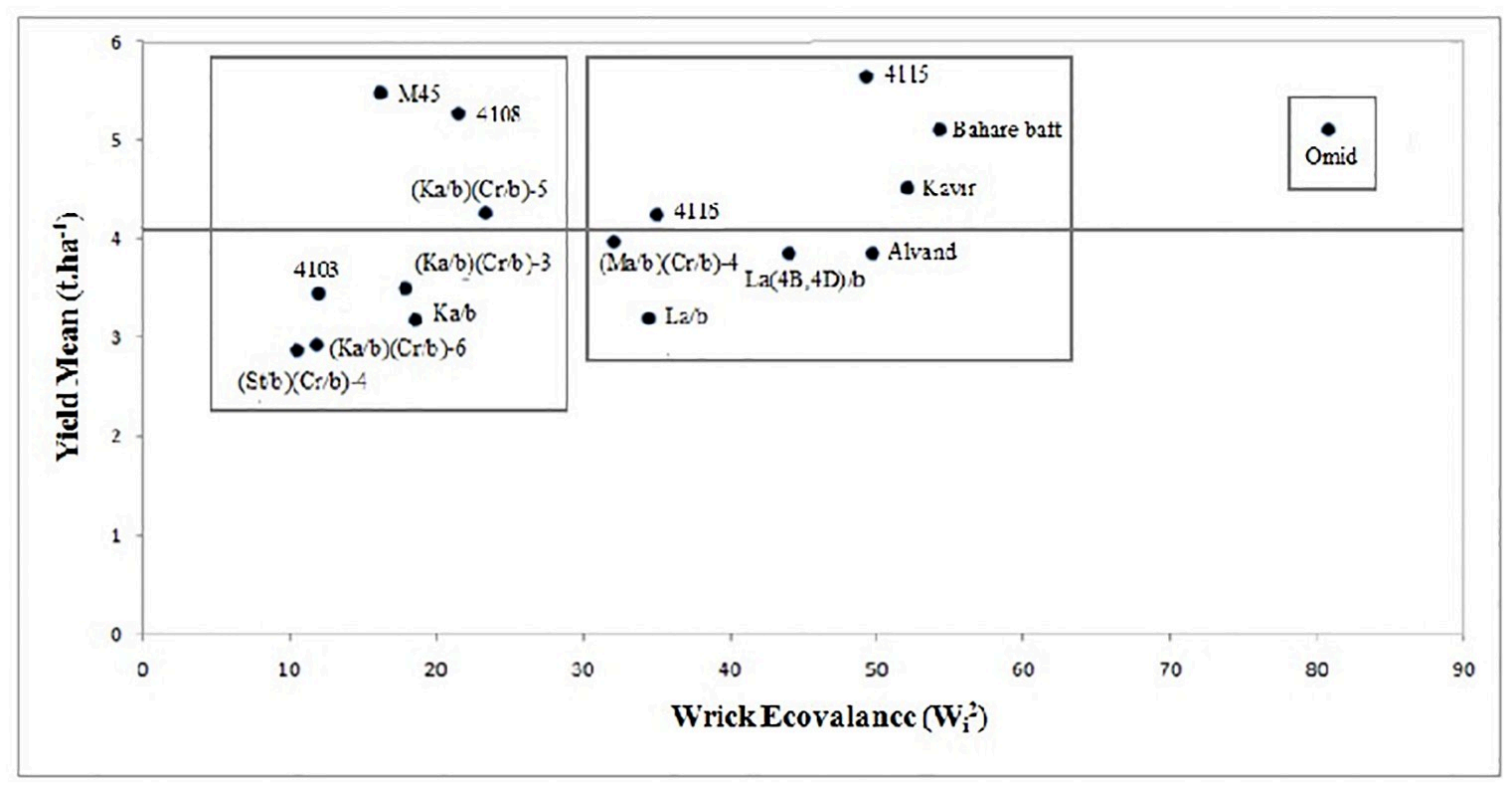
Biplot graph for three amphiploids of non-Iranian primary and combined primary tritipyrum lines, promising triticale lines and bread wheat varieties based on the mean of grain yield and Wrick ecovalance.

Based on the stability parameter of Wrick ecovalance (W^2^_i_) that gave exactly similar results to the Shukla’s stability variance (σ^2^_i_) values, the (St/b)(Cr/b)-4 combined primary tritipyrum line and Omid wheat with the lowest and highest value of these parameters were identified as the most stable and the most unstable genotypes, respectively (Table 1). The parameters of W^2^_i_ and σ^2^_i_ are representative of the contribution of each genotype in the GEI sum of squares. Cluster analysis results of three amphiploids showed the same grouping based on the W_i_^2^, σ_i_^2^ and the GEI sum of squares for each genotype. This is consistent with results from other studies which also reported the same grouping for W^2^_i_, σ^2^_i_ and the GEI sum of squares [72, 73]. The genotypes were divided into two stable and unstable groups (with two subgroups) based on the cluster analysis results of Wrick ecovalance and mean grain yield (Fig 4). The four bread wheat varieties (Omid, Alvand, Bahare baft, Kavir), three tritipyrum lines {La/b, La (4B,4D)/b, (Ma/b)(Cr/b)-4} and two triticale lines (4115 and 4116) were placed in the unstable group. The 4115 and 4116 lines and bread wheat varieties (Omid, Alvand and Kavir) showed specific adaptation to favorable environments with high ecovalance and higher yield than the mean, and Omid wheat, La/b, La (4B,4D)/b, (Ma/b)(Cr/b)-4 tritipyrum lines showed specific adaptation to unfavorable environments with a lower yield than the mean. In the first subgroup of stability, two tritipyrum lines {(Ka/b)(Cr/b)-6, (St/b)(Cr/b)-4} and 4103 triticale line with the lower yield and GEI sum of squares were placed, which indicated the weak general adaptation. The second subgroup included three primary tritipyrum lines {Ka/b, (Ka/b)(Cr/b)-3, (Ka/b)(Cr/b)-5} and two promising triticale lines (4108, M45) with average stability. The (Ka/b)(Cr/b)-5 tritipyrum line and two 4108, M45 triticale lines had relatively low GEI and higher yield than the mean, which is consistent with the results of Bakhshayeshi Geshlagh [74], Kebriyai et al. [75], and Zhiani et al. [76].

### AMMI method

It is important for breeding and cultivar recommendations to select genotypes that are stable across environments. The stability of genotypes is often assessed using AMMI biplots. Based on AMMI model (simultaneous analysis of additive main effects and multiplicative interaction effects), the effects of the environment, GEI, and three components of the first from the composition of the six principal components of interaction (IPC) were significant at P < 0.01 and the other three components were combined with the residual or noise (Table 2). Results of AMMI analysis showed that the first, second and third principal components (IPC1, IPC2, and IPC3) included 49.90%, 20.25%, and 19.34% of the interaction sum of squares, respectively. The F-test for IPC1, IPC2, and IPC3 was significant at P < 0.01. Thus, it was used from the AMMI3 model (Table 2), which is consistent with the results of Haji Mohammad Ali Jahromiet al. [77] and Temesgen et al. [78] in wheat. Erdemci et al. [79] reported the efficiency of this method in the detection of GEI. In plant breeding, experimental environments should indicate the cultivation areas so that GEI can be considered for when choosing the highly performing genotypes. A breeding program does not presently require including a large number of environments, but rather contains environments in which great variance can be observed [80]. The results indicated that 61.46% of the total sum of squares was attributable to GE interaction effects (Table 2).

**Table 2:** Variance analysis of grain yield of non-Iranian primary and combined primary tritipyrum lines, promising triticale lines and bread wheat varieties in seven different environments using AMMI method.

Source of variation	Degree of freedom	Sum of squares	Mean squares	F
Model	118	902.48	7.65	127.5**
Block (Replication in Environment)	14	1.69	0.28	4.67**
Genotype	16	93.94	5.87	1.00^n.s^
Environment	6	243.19	40.53	6.66**
Genotype × Environment	96	563.61	5.87	97.81**
IPCA1	21	281.27	13.39	223.17**
IPCA2	19	114.13	6.01	100.17**
IPCA3	17	108.99	6.41	106.83**
Residual	39	59.22	1.52	25.33
Error	224	14.53	0.06	-
Total	356	917.02	-	-

** and ^ns^: highly significant (α = 1%) and non-significant, respectively.

In AMMI model, the first three components explained 89.49% of the sum of the squares of the GE interactions (Table 2). In comparison with the regression method, in which, only 11.81% of the sum of the squares of the GE interactions was justified by the linear model. In the AMMI3 model, this contribution was about 7.6 times higher than the contribution of the linear regression component. Noise made up 10.51% of the sum of squares of the GE interactions in the AMMI3 model. As a result, GE interaction analysis with this model is more accurate and reliable than regression methods. The GEI makes it hard to choose the best performing and most stable genotypes. The large E and GEI in this study propose the probable existence of dissimilar mega-environments with various high-yielding genotypes [81]. The AMMI model’s superiority in describing a higher percentage of GEI compared to the linear regression model was also obvious in the other wheat studies [80].

In the AMMI model, the parameters of GE interactions for genotypes and environments are shown in S8 Table in S1 File. Also, the Eigen value and principal component values for genotypes and environments are given in S9 Table in S1 File. These values were used to evaluate the stability of genotypes and environments in biplot and also to calculate the stability parameters of AMMI model.

Cluster analysis results of genotypes and environments are shown based on the first three components of the AMMI model and mean yield. The middle horizontal line of these graphs shows the total mean. The genotypes and environments located on this line have a similar response in terms of the additive main effects (mean yield). The vertical axis in the middle of the graph has IPC = 0, which indicates the area of no interaction. Therefore, genotypes and environments on the vertical line have a similar response in terms of interactions. Genotypes and environments with high principal component scores (either plus or minus sign) have high interactions, those with values close to zero have low interactions. Genotypes and environments that have the same signs for principal components have positive interactions, while opposite signs give negative interactions. In general, AMMI and biplot analyses can help the breeder to have a comprehensive view of the genotypes, environments and GE interactions.

Cluster analysis of varieties and lines divided genotypes into three groups based on values of the first principal component (IPC1) and yield mean (S3 Fig). The Ka/b, La/b, La(4B,4D)/b, (Ma/b)(Cr/b)-4, (Ka/b)(Cr/b)-3 and (Ka/b)(Cr/b)-5 primary tritipyrum lines were placed in the first group with values of large and negative IPC1. The second group included 4108, 4103, 4115, 4116, M45 triticale lines, Omid, Alvand, Bahare baft and Kavir wheat varieties with values of positive IPC1. The third group included two primary tritipyrum lines {(Ka/b)(Cr/b)-6, (St/b)(Cr/b)-4} with average and negative IPC1. Also, cluster analysis of environments identified three groups based on values of the first principal component. The e_1_, e_2_, e_5_, and e_6_ with high and positive IPC1, e_4_ and e_7_ with negative IPC1 and e_3_ with high and negative IPC1 were placed in the first, second, and third groups, respectively.

The greater positive/negative IPCA scores, the more specifically adapted a genotype is to certain environments. The more IPCA scores close to zero, the more stable the genotype is over all environments tested [82]. Genotypes close to each other present similar performance, and those that are close to the environment indicate their better adaptation to that particular environment.

Pattern analysis based on IPC1 and means showed that M45 triticale line was the most stable and high yielding genotype with the lowest GEI and higher yield than the mean and 4116 triticale line had the second rank of stable and high yielding genotype (S3 Fig). Results showed that tested environments have a relatively high share in the GEI and the (Ka/b)(Cr/b)-5, (Ma/b)(Cr/b)-4 and La(4B,4D)/b tritipyrum lines had specific adaptation to e_3_, the 4103 and 4116 triticale lines to e_2_ and e_5_, the 4108, 4115 triticale lines, Kavir and Bahare baft wheat varieties to e_6_ (S3 Fig). The (Ka/b)(Cr/b)-6 and (St/b)(Cr/b)-4 tritipyrum lines showed specific adaptation to e_4_ and e_7_ (Sirjan), which is consistent with the results from other studies [36, 83] that showed the AMMI model is a useful tool for detection of GEI.

Cluster analysis divided the genotypes into three groups based on values of the second principal component (IPC2) and mean yields (S4 Fig). The first group included the 4108 and 4116 lines with the highest positive IPC2. The Ka/b, La/b, (Ka/b)(Cr/b)-3, (Ka/b)(Cr/b)-5, (Ka/b)(Cr/b)-6, (St/b)(Cr/b)-4 tritipyrum lines, 4115 triticale line, Omid and Bahare baft wheat varieties were placed in the second group with values of average and positive IPC2. The third group included La(4B,4D)/b, (Ma/b)(Cr/b)-4 tritipyrum lines, 4103, M45 triticale lines, Alvand and Kavir wheat varieties with negative IPC2. Cluster analysis of environments divided the environments into three groups based on the values of the second principal component (S4 Fig). The e_2_, e_3_,e_4_, and e_7_ with very low IPC2, e_1_ and e_5_ with high and negative IPC2, and e_6_ with high and positive IPC2 are placed in the first, second, and third groups, respectively. Since Bahare baft wheat had IPC2 close to zero and higher yields than the mean with the lowest GEI among the Ka/b, La/b, (Ka/b)(Cr/b)-6 tritipyrum lines and Bahare baft wheat, so they can be considered as the most stable genotypes. The e_2_, e_4_ and e_7_ had the lowest share in the expression of GEI.

Cluster analysis of genotypes identified three groups based on values of the third principal component (IPC3) and mean yields (S5 Fig). The (Ka/b)(Cr/b)-3, 4115 lines and Bahare baft wheat variety were placed in the first group with high and negative IPC3. The second group included (Ka/b)(Cr/b)-6, M45 lines and Omid wheat variety with positive IPC3. The Ka/b, La/b, La(4B,4D)/b, (Ma/b)(Cr/b)-4, (Ka/b)(Cr/b)-5, (St/b)(Cr/b)-4 tritipyrum lines, 4103, 4108, 4116 triticale lines and Alvand and Kavir wheat varieties were placed in the third group with IPC3 close to zero and the most stability. Also, cluster analysis of environments identified three groups based on values of the third principal component (S5 Fig). The 4108, 4116 triticale lines and Kavir wheat were identified as high yielding stable genotypes with IPC3 close to zero and higher yield than the mean. It is clear that a less portion of the interaction by the third and second component than the first component is the reason for the difference between the three biplots. So, the AMMI3 model used in this analysis calculated the stability statistics of EV_3_ and SIPC_3_ (S10 Table in S1 File). In the present study, the AMMI model exhibited that there was a more complex GEI, and it could not help graphical visualization of the genotypes in low dimensions, and then it is necessary to use a substitute method to GEI interpretation using AMMI parameters [81].

**Fig 5:**
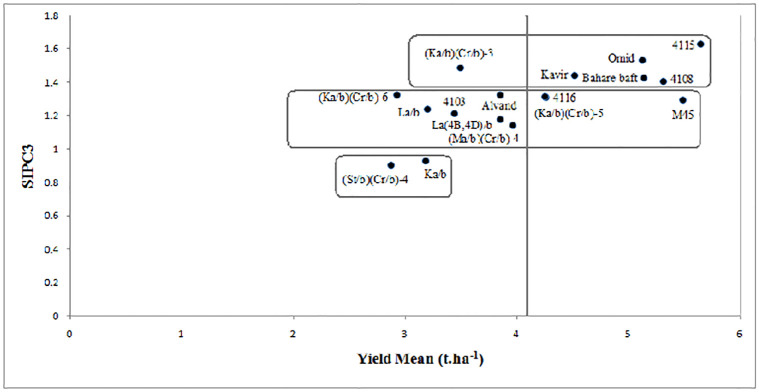
Biplot graph for three amphiploids of non-Iranian primary and combined primary tritipyrum lines, promising triticale lines and bread wheat varieties based on the mean of grain yield and SIPC_3_ stability parameter.

Cluster analysis of genotypes based on SIPC_3_ statistic (Fig 5) showed the Ka/b and (St/b)(Cr/b)-4 primary tritipyrum lines had a weak general adaptation with the lowest value of SIPC3 and lower yield than the mean. The M45, (Ka/b)(Cr/b)-5 and 4116 lines were identified as the most stable genotypes with low SIPC_3_ and higher yield than the mean, respectively. The Omid, Baharebaft, Kavir wheat varieties, (Ka/b)(Cr/b)-3, 4108 and 4115 lines were identified as the most unstable genotypes with the highest values of SIPC3.

Based on EV_3_ statistics, cluster analysis of genotypes (S6 Fig) divided the genotypes into three different groups. The Ka/b, La(4B,4D)/b, (Ma/b)(Cr/b)-4, (Ka/b)(Cr/b)-5 and (St/b)(Cr/b)-4 primary tritipyrum lines with the lowest values of EV3 were considered as the most stable genotypes. Thus, (Ka/b)(Cr/b)-5 primary tritipyrum line is selected as a desirable genotype with more stability and higher yield than the mean based on both stability statistics (EV_3_ and SIPC_3_).

**Fig 6:**
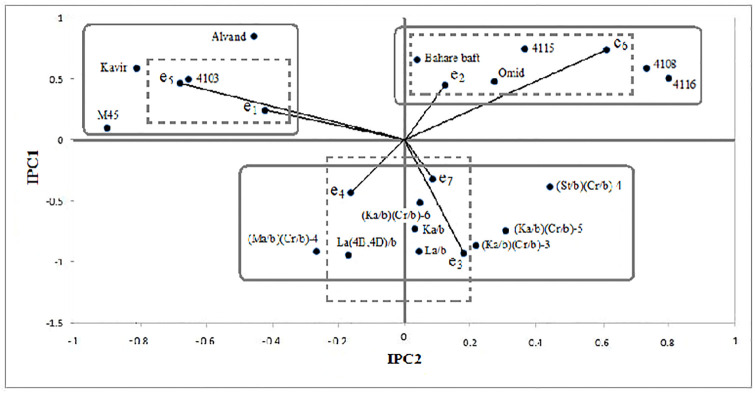
Biplot graph of first and second principal components for three amphiploids of non-Iranian primary and combined primary tritipyrum lines, promising triticale lines and bread wheat varieties and environments. (Interconnected and non-interconnected lines show grouping obtained from cluster analysis of genotypes and environments based on the amount of two first principal components, respectively. Horizontal and vertical lines pass from the first and second principal component points equal to zero, respectively). e_1_: Kerman (normal) and fourth crop year, e_2_: Kerman (normal) and second crop year, e_3_: Kerman (normal) and third crop year, e_4_: Sirjan (normal) and fourth crop year, e_5_: Neyriz (normal) and first crop year, e_6_: Kerman (normal) and first crop year, and e_7_: Sirjan (salinity) and fourth crop year.

The biplot of first and second principal components for genotypes and environments (Fig 6) explained 70.16% of the GEI information. This biplot showed three distinct groups of genotypes. The 4108, 4115, 4116 triticale lines, Omid and Bahare baft wheat varieties were placed in the first group with positive values for both IPC. The second group included Ka/b, La/b, La(4B,4D)/b, (Ma/b)(Cr/b)-4, (Ka/b)(Cr/b)-3, (Ka/b)(Cr/b)-5, (St/b)(Cr/b)-4 and (Ka/b)(Cr/b)-6 primary tritipyrum lines. The third group included 4103, M45 triticale lines, Alvand and Kavir wheat varieties with opposite IPC values. Cluster analysis of environments identified three groups based on the values of the first and second principal components (Fig 6). The results showed that two tritipyrum lines {(Ka/b)(Cr/b)-6, (St/b)(Cr/b)-4} and Bahare baft wheat have general stability. According to this, triticale lines {4103, 4115, 4108, 4116} and wheat varieties {Omid, Alvand and Kavir} had high interactions and primary tritipyrum lines {Ka/b, La/b, La(4B,4D)/b, (Ka/b)(Cr/b)-3, (Ka/b)(Cr/b)-5} showed average interactions. Also, the M45 triticale line was identified as a stable genotype regarding the larger contribution of the first component than the second component. According to the results, the 4103 triticale line and Alvand and Kavir wheat varieties had specific adaptation to e_1_ and e_5_ and the 4108, 4115 and 4116 triticale lines showed specific adaptation to e_6_. Also, La (4B,4D)/b and (Ma/b)(Cr/b)-4 tritipyrum lines had specific adaptation to e_4_ (Fig 6). Environmental vectors indicate a positive correlation between e_1_ and e_3_ with e_4_, e_2_ with e_6_ and e_4_ with e_5_ and e_7_ in the expression of the interactions. But there was no correlation between e_6_ with e_3_ and e_7_ (Fig 6).

The studies showed that the most accurate model for AMMI can be predicted using the first two IPC. The factors similar to germplasm diversity, crop type and environmental conditions will affect the complexity degree of the best predictive model [84]. Tarinejad and Abedi [85] used stability analysis methods of Wrick’s ecovalence, stability variance of Shukla, Eberhurt and Russell, AMMI and GCE biplot to determine the stability of grain yield in bread wheat and the introduction of stable genotypes.

Many investigations have assessed wheat genotypes and the AMMI model has been specified for proper identification of genotypes with general and specific adaptability to diverse environments [86]. In this study, stability evaluation of 17 varieties and lines of hexaploid amphiploid with the multivariate method of AMMI showed that tritipyrum and triticale lines had more stability and adaptability than Iranian wheat varieties. The (Ka/b)(Cr/b)-5 combined primary tritipyrum line with a higher yield than mean, had good general adaptation. Although the response of wheat varieties was in the range of instability up to poor stability, triticale lines had the response of average stability up to poor stability. So, the primary tritipyrum lines can be considered as a new plant with a higher potential of stability than wheat and triticale in the expression of general adaptation. In this order, Yadav et al. [87] mentioned that AMMI approach is an effective method to delineate GEI and stability of barley (*Hordeum vulgare* L.) genotypes under northern Indian Shivalik hill conditions.

Dimitrijvic et al. [88] suggested the most stable genotypes in the study of stability of yield components in wheat using the AMMI method. Solomon et al. [89], in analyzing the genotypic responses of 23 durum wheat genotypes to 12 environments by AMMI method, reported a significant interaction for the first two principal components and, on average 94% of the total interaction squares was explained by the first principal component. Moreover, they suggested the superiority of the AMMI method compared with the Eberhart and Russell regression method.

### Path analysis of GEI (Tai method)

Analysis of GEI with Path coefficient analysis or Tai’s Method [54] is based on the coefficient of correlation analysis. Thus, the correlation coefficients were calculated between yield and its components for varieties and lines (S11 Table in S1 File). None of the genotypes showed significant correlation between yield and grain number per spike except Alvand and Omid wheat varieties, so grain number per spike was the least important component of yield for evaluated genotypes. Correlation coefficients of spike number with yield were significant in most varieties and lines than the grain weight with yield. In varieties and lines with a higher yield than mean, there is a negative and significant correlation between yield and spike number (S11 Table in S1 File) and the highest correlation coefficient observed between grain yield and spike number in the (St/b)(Cr/b)-4 tritipyrum line and Kavir wheat variety. The 4115 triticale line had the highest correlation coefficient between grain weight and grain yield (r = -0.62**). The high correlation between any spike number and grain weight with yield showed that these were more important components to determine grain yield of varieties and lines. Correlation of yield components with each other had considerable differences in different varieties and lines (S11 Table in S1 File). The correlation coefficient between spike number and grain number per spike was positive in all varieties and lines except primary tritipyrum lines {(Ma/b)(Cr/b)-4, La(4B,4D)/b, (Ka/b)(Cr/b)-3, (Ka/b)(Cr/b)-5, (Ka/b)(Cr/b)-6} and was significant in (Ma/b)(Cr/b)-4, (Ka/b)(Cr/b)-3, 4108, 4116, 4115, M45 lines and Kavir wheat cultivar. The highest and lowest correlation between spike number and grain number per spike were observed in M45 triticale line and (Ka/b)(Cr/b)-6 tritipyrum line, respectively (S11 Table in S1 File). The correlation coefficient between spike number and grain weight was significant and positive in all varieties and lines except Ka/b primary tritipyrum line. The highest and lowest value of positive and significant correlation between spike number and grain weight belonged to the 4116, M45 triticale lines and La/b primary tritipyrum line, respectively. The correlation coefficient was negative between grain number per spike and grain weight of (Ma/b)(Cr/b)-4, (Ka/b)(Cr/b)-3, (Ka/b)(Cr/b)-5 and (Ka/b)(Cr/b)-6 primary tritipyrum lines.

To determine the contribution of yield components in GEI, the results of path analysis are listed in S12 and S13 Tables in S1 File. The highest and lowest direct effect are related to spike number on grain yield (a_4_) and grain number per spike (a_5_), respectively. The highest direct effect of the spike number on grain yield (a_4_) belonged to Kavir wheat, triticale lines {4116, M45, 4103} and tritipyrum line {(Ka/b)(Cr/b)-3, (St/b)(Cr/b)-4, La/b} (S10 Table in S1 File). Also, Omid, Alvand and Bahare baft wheat varieties showed the highest direct effect of grain number per spike on grain yield (a_5_) (S12 Table in S1 File). The direct effect of grain weight on grain yield (a_6_) of 4116 triticale was higher than the other genotypes but, tritipyrum lines} (Ma/b)(Cr/b)-4, (Ka/b)(Cr/b)-5, (Ka/b)(Cr/b)-6, (St/b)(Cr/b)-4} and triticale lines) M45, 4108 (had the lowest value (S10 Table in S1 File). Stability response of tritipyrum lines} Ka/b, La/b, La (4B,4D)/b, (Ma/b)(Cr/b)-4, (Ka/b)(Cr/b)-3, (Ka/b)(Cr/b)-5, (Ka/b)(Cr/b)-6, (St/b)(Cr/b)-4}, triticale lines {4103, 4108, 4115, M45} and Kavir wheat related to genotypic component of GEI for spike number (S13 Table in S1 File). Among the triticale lines (M45, 4108, 4115), (Ka/b)(Cr/b)-5 tritipyrum and Kavir wheat with the high yield and relatively stable, Kavir wheat had the highest genotypic component of spike number (V_1_). The highest genotypic component of grain weight (V_3_) belonged to La/b and 4116. The highest genotypic component of grain number per spike (V_2_) belonged to Omid, Alvand and Bahare baft wheat varieties and these varieties potentially have a good and stable yield. The highest contribution of GEI is affected by grain number per spike in the unstable lines and varieties of these three amphiploids (S13 Table in S1 File). Therefore, plant characteristics that are developed at this stage are strongly affected and reduce the yield and stability of the cultivars and lines when cultivated in unfavorable agronomic or climatic conditions, because these conditions have the greatest effects on the growth and characterization of the plant during growth stages. Omid and Alvand wheat varieties clearly indicate these results. According to the results of Ibrahim et al. [90], generally, Tai’s stability method was facilitated the visual comparison and identification of superior genotypes, thereby supporting decisions grain sorghum genotypes for different environments.

The highest environmental sensitivity was observed in the flowering and pollination stage (r_2_) for most of the environments. e_1_, e_5_ and e_3_ had the highest environmental sensitivity in the r_3_ stage. In other words, those genotypes which have a high environmental sensitivity will have a considerable reduction of yield in this stage. Tillering stage (r_1_) had the least sensitivity in all environments, and this means that environmental stress did not have a significant effect on grain yield at this stage. The lowest value of r_3_ was observed in e_4_ (Sirjan). Based on the Tai method (S14 Table in S1 File) at the tillering stage, the highest genotypic component of V_1_ was related to the most stable wheat cultivar (Kavir) with a high yield. The 4116 unstable triticale line had a very high V_3_. Since V_3_ shows the correlation between grain weight and grain yield and this correlation is high in unstable lines and cultivars. Therefore, the V_3_ parameter is not an appropriate criterion for selection instability analysis. Due to the high variability of the r_2_ component than r_1_ and r_3_ components, it seems that different genotypes have more sensitivity to environmental conditions in the flowering and seed formation stage (S14 Table in S1 File). Therefore, the selection of stable genotypes based on the genotypic component of V_2_ has less reliability. The genotypic component of V_1_ can be introduced as a better criterion for the selection of stable and high yielding genotypes due to the low variability of r_1_ environmental components and high yield correlation with spike number than the other yield components. In the evaluation of environments, stress at r_2_ stage (flowering and pollination) had a greater impact on yield, which is consistent with the results of Mohammadinejad and Rezaei [91]. In contrast to our results, Askarinia et al. [60] in a stability analysis of wheat genotypes via Thai method found that the genotypic component of 1000-grain weight is the most important genotypic component affecting yield and stability and also, the sensitivity of grain weight to environmental changes is less than the other two components (spikes number and grain number per spike). Mohammadi et al. [92] reported that higher grain yields are correlated with higher kernel weight, which resulted from early flowering, and therefore, more emphasis should be given to these features for the improvement of wheat yield under rainfed condition.

**Fig 7:**
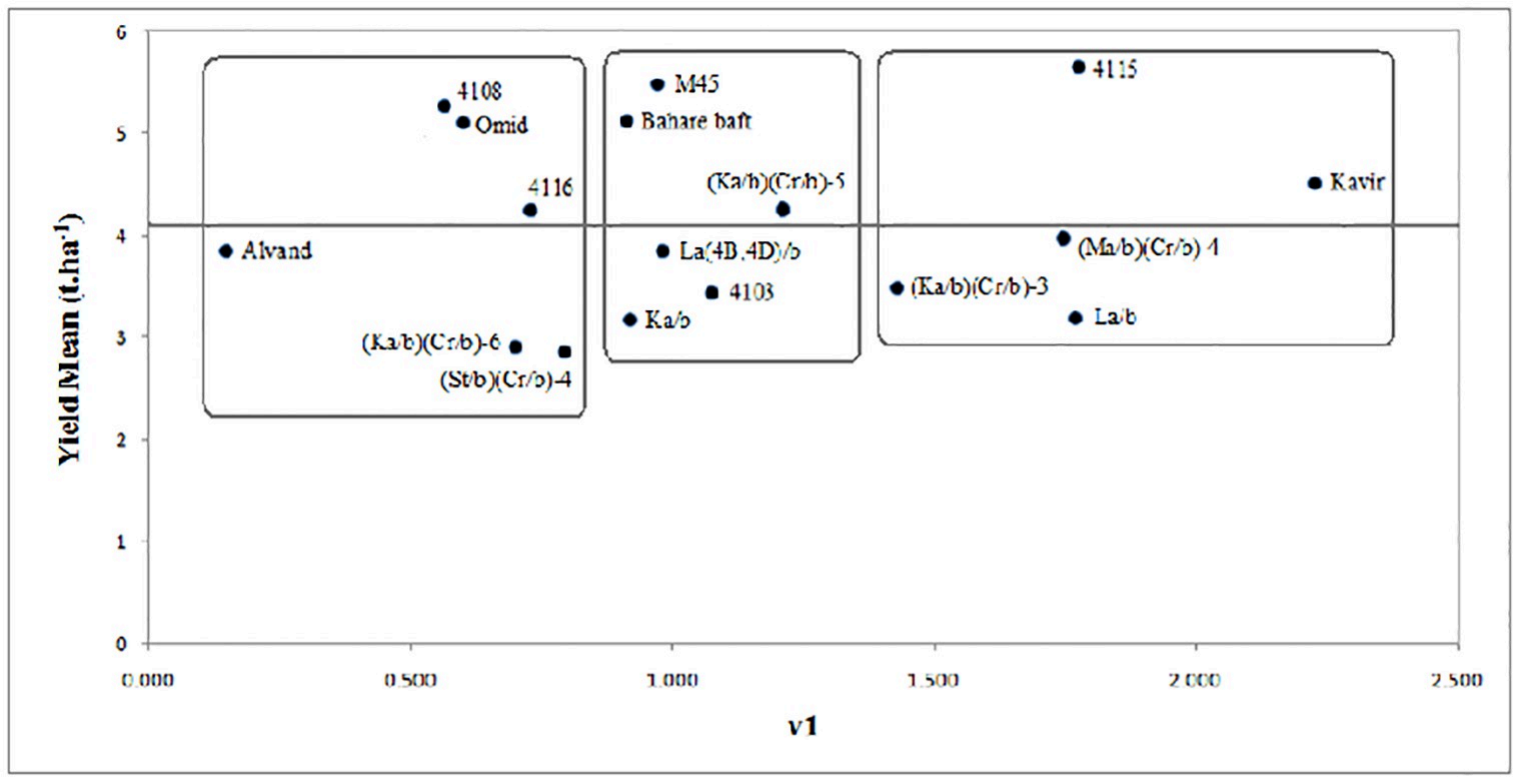
Biplot graph for three amphiploids of non-Iranian primary and combined primary tritipyrum lines, promising triticale lines and bread wheat varieties based on the mean of grain yield and the V_1_ parameter of the Tai method.

Cluster analysis results (Fig 7) divided the varieties and lines into three groups based on the genetic component of V_1_. Primary tritipyrum lines} La/b, (Ma/b)(Cr/b)-4, (Ka/b)(Cr/b)-3}, 4115 triticale line and Kavir wheat were placed in the first group with high stability. The second group included the primary tritipyrum lines {Ka/b, La (4B,4D)/b, (Ka/b)(Cr/b)-5}, triticale lines {4103, M45} and Bahare baft wheat with average stability. Two combined primary tritipyrum lines} (Ka/b)(Cr/b)-6, (St/b)(Cr/b)-4}, two triticale lines and two wheat varieties {Omid, Alvand} were placed in the third group which showed the in stability response. The Kavir wheat variety and 4115 triticale line were the most stable genotypes, respectively, with higher yields than mean and high V_1_. Alvand wheat was the most unstable genotype with low V_1_ and lower yield than the mean (Fig 7).

Based on the results of Tai method, the primary and combined primary tritipyrum lines demonstrated higher stability in comparison with Iranian wheat varieties and promising triticale lines. These results not only confirm the results of various stability analysis methods, but also provide insights into the different components of GEIs. In other words, determining the contribution of each stage of growth and development in GEIs may contribute to adopt targeted breeding methods and agricultural practices for achieving the maximum production capacity of different crop cultivars.

### Correlation of stability parameters

Since the number of the stability parameters is increasing, determination of the correlation between the parameters can be effective in reducing the number of parameters. One of the ways to determine the correlation between stability parameters is the use of Spearman’s rank correlation that has been used by many researchers [34]. In this study, a positive and significant correlation was observed between the most stable parameters of univariate and multivariate (S15 Table in S1 File). This outcome agrees with findings reported by Ahmadi et al. [93], Pour-Aboughadareh et al. [94], and Vaezi et al. [95]. The outcomes indicated that AMMI-based stability statistics had a significant positive correlation with each other and also with most parametric statistics. Stability parameters of linear regression, deviation from the linear regression, Wrick ecovalance, and Shukla stability variance indicated a high and negative correlation with yield. While a positive and significant correlation was shown between mean grain yield and determination coefficient. The second criterion of Eberhart and Russell’s stability (S^2^_di_) was significantly (P < 0.01) and positively correlated with W^2^_i_, σ^2^_i_ and S^2^_i_. A rank correlation coefficient of 1.0 was found between W^2^_i_ and σ^2^_i_. This indicated that these two procedures were equivalent for ranking purposes. Dissimilar results were observed by Anley et al. [96]. Also, in the AMMI analysis was observed a positive and significant correlation between the stability statistics (SIPC_3_ and EV_3_), components of first, second and third (S15 Table in S1 File). Baxevanos et al. [97] evaluated 36 cotton genotypes in 20 regions of Greece, Spain and Turkey for 6 consecutive years and suggested a significant correlation of σ^2^_i_ with S^2^_di_ and AMMI1. Moreover, grain yield had no correlation with S^2^_di_ and σ^2^_i_, but revealed a correlation with regression coefficient and AMMI statistics in some years. In the study by Karimzadeh et al. [98], four parameters including SIPC_4_, AMGE_4_, ASV and EV_4_ were utilized for stability evaluation of 10 corn hybrids. EV_4_, SIPC_4_ and ASV parameters had no correlation with each other. ASV parameter revealed highly significant positive correlation with Huehn’s S^2^ nonparametric statistics and Wricke ecovalence. The slope of Finlay and Wilkinson [40] regression line also did not show a significant correlation with any of the other parameters. Correlations between estimates of adaptability, stability, and yield parameters help to better predict the behavior of the assessed genotypes. According to the correlation matrix, the parametric stability methods used in this study disclosed that these could be used to assess the response of genotypes to changing environments.

## Conclusion

The reactions of genotypes to unstable abiotic factors can be evaluated by research across several years and/or in diverse localization. In this study, different methods of yield stability showed many similar results. The study has clearly proven that the AMMI model can summarize patterns and relationships of genotypes and environments successfully. And thus, the information from the AMMI model could be important to release genotypes to target environments based on their responses. Combined primary tritipyrum lines had the most yield stability and greater adaptability than the promising triticale lines and Iranian wheat varieties. The (Ka/b)(Cr/b)-5 and M45 were the most stable tritipyrum and triticale genotypes, respectively (Table 3). Thus, (Ka/b)(Cr/b)-5 new tritipyrum line and M45 triticale line with the mean yield of 4.26 and 5.48 (t.ha^-1^), respectively, can be introduced as the high yielding and the most stable genotypes for many poor and saline soil conditions. Also, complementary agronomic experiments may release a new grain crop of triticale and new pasture line of combined primary tritipyrum for grain and forage. Moreover, the combined tritipyrum line can be used in bread wheat breeding program for producing salt-tolerant wheat genotype/s.

**Table 3:** Summary of different stability parameters for three hexaploid amphiploids including non-Iranian primary and combined primary tritipyrum lines, promising triticale lines, and bread wheat varieties in different environmental conditions.

Method		Stable varieties and lines	Unstable varieties and lines
Univariate	Linear regression method	Ka/b, La/b, (Ma/b)(Cr/b)-4, (Ka/b)(Cr/b)-3, (Ka/b)(Cr/b)-5, (Ka/b)(Cr/b)-6, (St/b)(Cr/b)-4, 4103, 4108, 4116 and M45	La (4B,4D)/b, 4115, Omid, Alvand, Kavir and Baharebaft
	Wrick ecovalance (W^2^_i_) and Shukla stability variance (σ^2^_i_)	(Ka/b)(Cr/b)-6, (St/b)(Cr/b)-4, Ka/b, (Ka/b)(Cr/b)-3, (Ka/b)(Cr/b)-5, 4108, 4103 and M45	La/b, La (4B,4D)/b, (Ma/b)(Cr/b)-4, 4115, 4116, Omid, Alvand, Kavir and Baharebaft
Multivariate (AMMI method)	First principal component (IPC_1_)	(Ka/b)(Cr/b)-6, (St/b)(Cr/b)-4, (Ka/b)(Cr/b)-5, 4116, 4103 and M45	Ka/b, La/b, La(4B,4D)/b, (Ma/b)(Cr/b)-4, (Ka/b)(Cr/b)-3, 4115, 4116, Omid, Alvand, Kavir & Baharebaft
	Second principal component (IPC_2_)	Ka/b, La/b, (Ka/b)(Cr/b)-3, (Ka/b)(Cr/b)-5, (Ka/b)(Cr/b)-6, (St/b)(Cr/b)-4, 4115, Omid, and Baharebaft	La(4B,4D)/b, (Ma/b)(Cr/b)-4, 4103, 4116, 4108, M45, Alvand and Kavir
	SIPC_3_ stability statistics	)St/b)(Cr/b)-4, La/b, La(4B,4D)/b, (Ma/b)(Cr/b)-4, (Ka/b)(Cr/b)-5, (Ka/b)(Cr/b)-6, 4116, 4103, M45 and Alvand	(Ka/b)(Cr/b)-3, 4108, 4115, Omid, Kavir and Baharebaft
	EV_3_ stability statistics	Ka/b, La(4B,4D)/b, (Ma/b)(Cr/b)-4, (Ka/b)(Cr/b)-5, (St/b)(Cr/b)-4, La/b, (Ka/b)(Cr/b)-3, 4103 and Alvand	(Ka/b)(Cr/b)-6, 4108, 4115, 4116, M45, Omid, Kavir and Baharebaft
Tai method	V_1_ genotypic component	La/b, (Ma/b)(Cr/b)-4, (Ka/b)(Cr/b)-3, Ka/b, La(4B,4D)/b, (Ka/b)(Cr/b)-5, 4116, 4103, M45, Kavir and Baharebaft	(Ka/b)(Cr/b)-6, (St/b)(Cr/b)-4, 4108, 4116, Omid and Alvand

## Supporting information

S1 Fig Mean comparison of grain yield for non-Iranian primary and combined primary tritipyrum lines, promising triticale lines, and bread wheat varieties in different environments. Means with similar letter(s) are not significantly different (α = 5%), using Duncan’s new multiple range test. e_1_: Kerman (normal) and fourth crop year, e_2_: Kerman (normal) and second crop year, e_3_: Kerman (normal) and third crop year, e_4_: Sirjan (normal) and fourth crop year, e_5_: Neyriz (normal) and first crop year, e_6_: Kerman (normal) and first crop year, and e_7_: Sirjan (salinity) and fourth crop year.
(PDF)

S2 Fig Biplot graph for non-Iranian primary and combined primary tritipyrum lines, promising triticale lines, and bread wheat varieties based on the mean of grain yield and linear regression coefficient of Eberhart and Russell. (Vertical line passes through the point of mean grain yield).
(PDF)

S3 Fig Biplot graph of mean of grain yield and the stability parameter of first principal component of the genotypes and environments. (Square and oval shapes show grouping obtained from cluster analysis of genotypes and environments based on the first principal component, respectively. Horizontal and vertical lines pass from the mean yield and first principal component points equal to zero, respectively). e_1_: Kerman (normal) and fourth crop year, e_2_: Kerman (normal) and second crop year, e_3_: Kerman (normal) and third crop year, e_4_: Sirjan (normal) and fourth crop year, e_5_: Neyriz (normal) and first crop year, e_6_: Kerman (normal) and first crop year, and e_7_: Sirjan (salinity) and fourth crop year.
(PDF)

S4 Fig Biplot graph of mean of grain yield and second principal component of genotypes and environments. (Interconnected and non-interconnected lines show grouping obtained from cluster analysis of genotypes and environments based on the second principal component, respectively. Horizontal and vertical lines pass through the mean yield and second principal component points equal to zero, respectively). e_1_: Kerman (normal) and fourth crop year, e_2_: Kerman (normal) and second crop year, e_3_: Kerman (normal) and third crop year, e_4_: Sirjan (normal) and fourth crop year, e_5_: Neyriz (normal) and first crop year, e_6_: Kerman (normal) and first crop year, and e_7_: Sirjan (salinity) and fourth crop year.
(PDF)

S5 Fig Biplot graph of mean of grain yield and stability parameter of third principal component of genotypes and environments. (Interconnected and non-interconnected lines show grouping obtained from cluster analysis of genotypes and environments based on the third principal component, respectively. Horizontal and vertical lines pass through the yield and third principal component points equal to zero, respectively). e_1_: Kerman (normal) and fourth crop year, e_2_: Kerman (normal) and second crop year, e_3_: Kerman (normal) and third crop year, e_4_: Sirjan (normal) and fourth crop year, e_5_: Neyriz (normal) and first crop year, e_6_: Kerman (normal) and first crop year, and e_7_: Sirjan (salinity) and fourth crop year.
(PDF)

S6 Fig Biplot graph for three amphiploids of non-Iranian primary and combined primary tritipyrum lines, promising triticale lines, and bread wheat varieties based on the mean of grain yield and EV_3_ stability parameter. (PDF)

S1 File (PDF)
