# Supplementary figures and images for: Evaluation of grain yield stability of tritipyrum as a novel cereal in comparison with triticale lines and bread wheat varieties through univariate and multivariate parametric methods
Source: PLoS One. 2022 Sep 29;17(9):e0274588. doi: 10.1371/journal.pone.0274588 (PMC9703957; doi:10.1371/journal.pone.0274588)

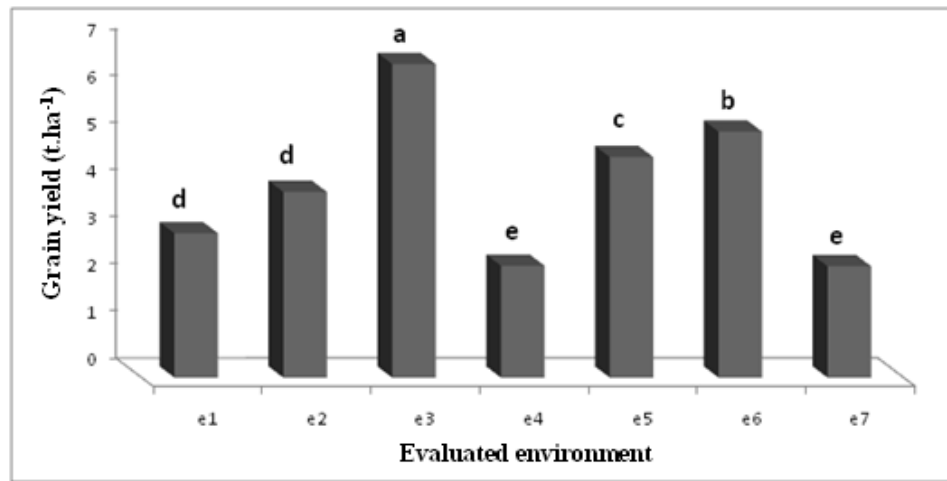

Supplement: S1 Fig — Means with similar letter(s) are not significantly different (α = 5%), using Duncan’s new multiple range test. e1: Kerman (normal) and fourth crop year, e2: Kerman (normal) and second crop year, e3: Kerman (normal) and third crop year, e4: Sirjan (normal) and fourth crop year, e5: Neyriz (normal) and first crop year, e6: Kerman (normal) and first crop year, and e7: Sirjan (salinity) and fourth crop year. (PDF) [file pone.0274588.s001.pdf]

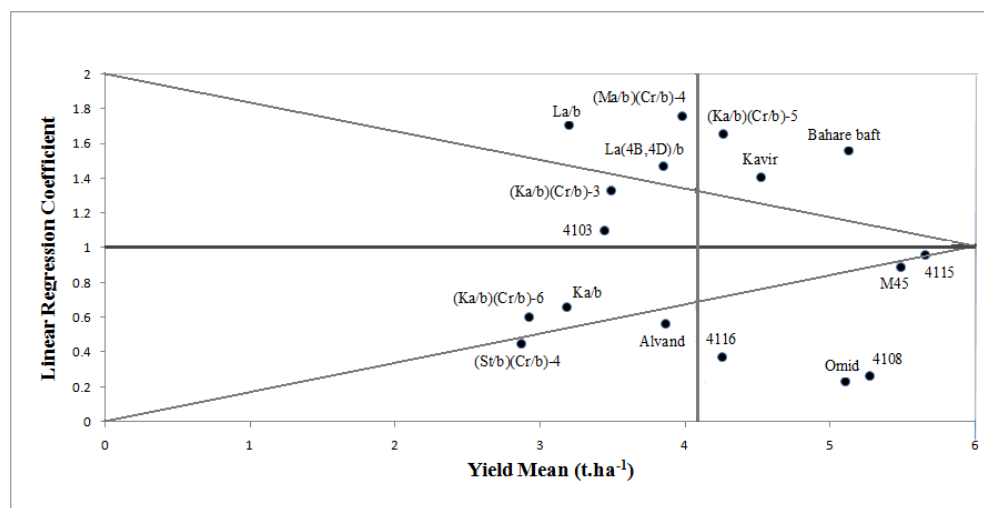

Supplement: S2 Fig — (Vertical line passes through the point of mean grain yield). (PDF) [file pone.0274588.s002.pdf]

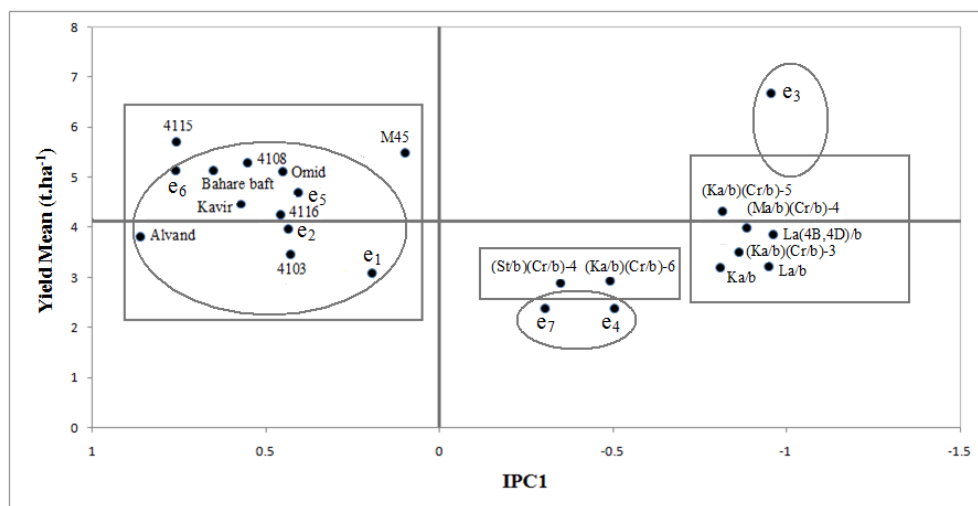

Supplement: S3 Fig — (Square and oval shapes show grouping obtained from cluster analysis of genotypes and environments based on the first principal component, respectively. Horizontal and vertical lines pass from the mean yield and first principal component points equal to zero, respectively). e1: Kerman (normal) and fourth crop year, e2: Kerman (normal) and second crop year, e3: Kerman (normal) and third crop year, e4: Sirjan (normal) and fourth crop year, e5: Neyriz (normal) and first crop year, e6: Kerman (normal) and first crop year, and e7: Sirjan (salinity) and fourth crop year. (PDF) [file pone.0274588.s003.pdf]

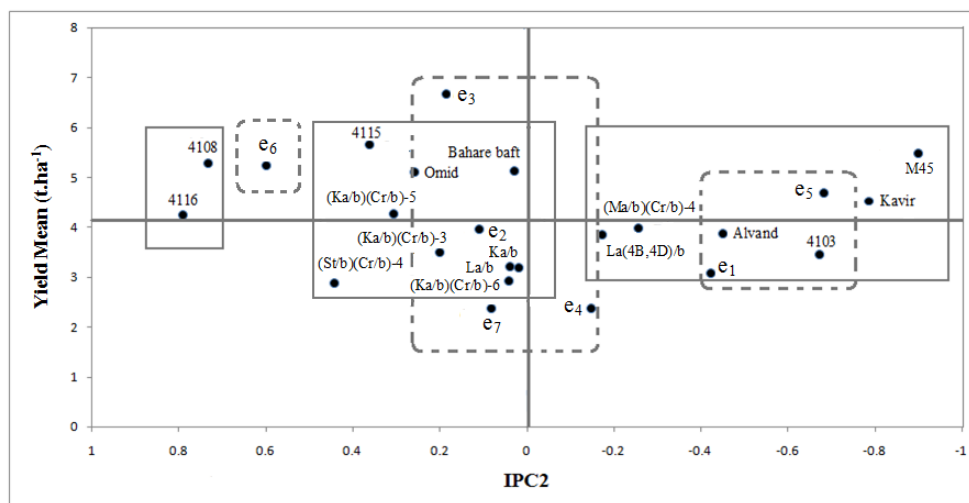

Supplement: S4 Fig — (Interconnected and non-interconnected lines show grouping obtained from cluster analysis of genotypes and environments based on the second principal component, respectively. Horizontal and vertical lines pass through the mean yield and second principal component points equal to zero, respectively). e1: Kerman (normal) and fourth crop year, e2: Kerman (normal) and second crop year, e3: Kerman (normal) and third crop year, e4: Sirjan (normal) and fourth crop year, e5: Neyriz (normal) and first crop year, e6: Kerman (normal) and first crop year, and e7: Sirjan (salinity) and fourth crop year. (PDF) [file pone.0274588.s004.pdf]

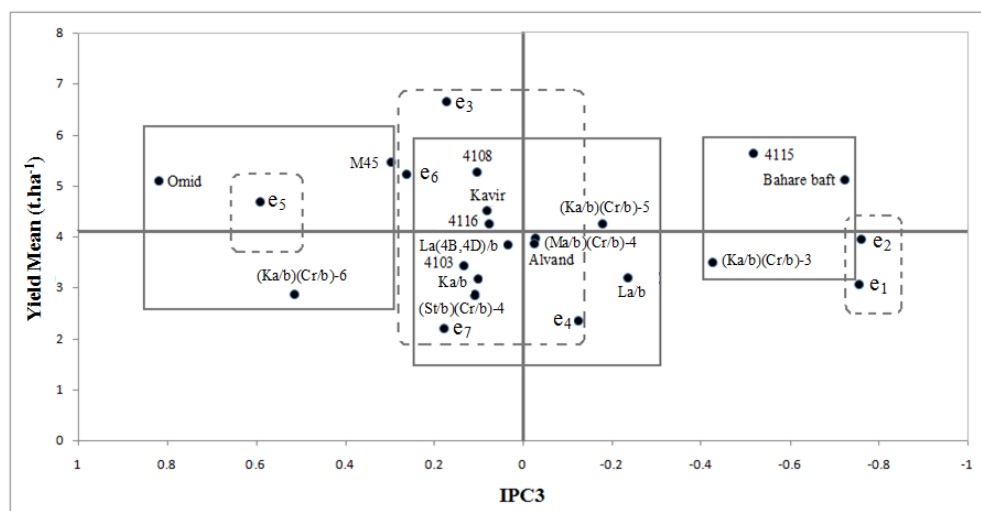

Supplement: S5 Fig — (Interconnected and non-interconnected lines show grouping obtained from cluster analysis of genotypes and environments based on the third principal component, respectively. Horizontal and vertical lines pass through the yield and third principal component points equal to zero, respectively). e1: Kerman (normal) and fourth crop year, e2: Kerman (normal) and second crop year, e3: Kerman (normal) and third crop year, e4: Sirjan (normal) and fourth crop year, e5: Neyriz (normal) and first crop year, e6: Kerman (normal) and first crop year, and e7: Sirjan (salinity) and fourth crop year. (PDF) [file pone.0274588.s005.pdf]

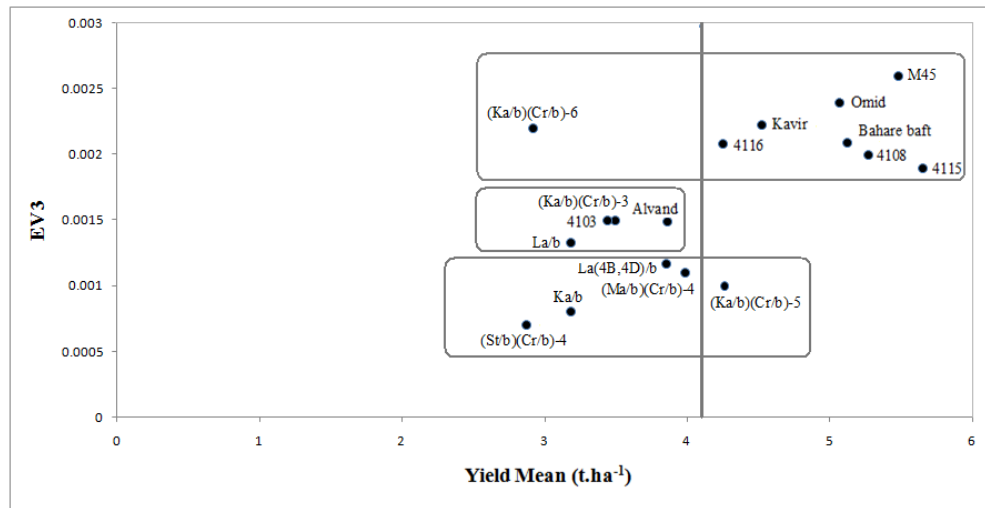

Supplement: S6 Fig — (PDF) [file pone.0274588.s006.pdf]
