# Supplementary material for: Evaluation of grain yield stability of tritipyrum as a novel cereal in comparison with triticale lines and bread wheat varieties through univariate and multivariate parametric methods
Source: PLoS One. 2022 Sep 29;17(9):e0274588. doi: 10.1371/journal.pone.0274588 (PMC9703957; doi:10.1371/journal.pone.0274588)
Supplement: S1 File — (PDF) [file pone.0274588.s007.pdf]

S1 Table. Different genotypes of cereals including non-Iranian primary and combined primary tritipyrum lines, promising triticale lines, and bread wheat varieties.

Code of the genotypes	Amphiploid type	Code of lines and varieties	Number and chromosomal formula
1	Primary tritipyrum lines	Karim/*Thinopyrumbessarabicum*=Ka/b	$AABBE^bE^b$, 2n=6x=42
2		Langdon/*Thinopyrumbessarabicum*=La/b	
3		Langdon(4B/4D)/*Thinopyrumessarabicum*=La(4B/4D)/b	
4	Combined primary tritipyrum lines	Macoun/*Th. bessarabicum*×Creso/*Th.bessarabicum*=(Ma/b)×(Cr/b)-4	$AABBE^bE^b$, 2n=6x=42
5		Karim/*Th. bessarabicum*×Creso/*Th. Bessarabicum*=(Ka/b)×(Cr/b)-3	
6		Karim/*Th. bessarabicum*×Creso/*Th. bessarabicum*=(Ka/b)×(Cr/b)-5	
7		Karim/*Th. bessarabicum*×Creso/*Th. Bessarabicum*=(Ka/b)×(Cr/b)-6	
8		Stewart/*Th .bessarabicum*×Creso/*Th.bessarabicum*=(St/b)×(Cr/b)-4	
9	Promising triticale lines	Triticale 4103	AABBRR, 2n=6x=42
10		Triticale 4108	
11		Triticale 4115	
12		Triticale 4116	
13		Triticale M45	
14	Iranian bread wheat varieties	Omid	AABBDD, 2n=6x=42
15		Alvand	
16		Baharebaft	
17		Kavir	

S2 Table. Types of experiments in different environments and cropping years.

Experiments Environments)	Location of experiments	Code of the Environments	Crop year
1	Kerman (Normal)	e_1	Fourth crop year
2	Kerman (Normal)	e_2	Second crop year
3	Kerman (Normal)	e_3	Third crop year
4	Sirjan (Normal)	e_4	Fourth crop year
5	Neyriz (Normal)	e_5	First crop year
6	Kerman (Normal)	e_6	First crop year
7	Sirjan (Salinity, Ec=15 $dS.m^{-1}$)	e_7	Fourth crop year

S3 Table. Bartlett's test based on chi-square for grain yield and other agronomic characteristics of non-Iranian primary and combined primary tritipyrum lines, promising triticale lines, and bread wheat varieties under different environmental conditions.

Traits	Chi-square (X^2)
Grain yield ($t.ha^{-1}$)	$8.25^{n.s}$
Harvest index (%)	$8.11^{n.s}$
1000-grain weight (g)	$11.38^{n.s}$
Grain number per spike	$10.64^{n.s}$
Spikelet number per spike	$7.71^{n.s}$
Spike number Per plant	$7.23^{n.s}$
Spike length (cm)	$5.25^{n.s}$
Tiller number per plant	$12.02^{n.s}$
Plant height (cm)	$9.06^{n.s}$
Day to heading	$44.28^{n.s}$

^{ns}: non-significant.

S4 Table. Combined analysis of variance for grain yield and other agronomic characteristics of non-Iranian primary and combined primary tritipyrum lines, promising triticale lines, and bread wheat varieties under different environmental conditions.

Source of variation	Degrees of freedom	Grain yield	Harvest index	1000-grain weight	Grain number per spike	Spikelet number per spike	Spike number Per plant	Spike length	Tiller number per plant	Plant height	Day to heading
Environment (E)	6	121.64**	1261.9**	5230.19**	923.24**	181.32**	1840.97**	393.31**	2697.39**	5719.27**	7379.87**
Error 1	14	0.36*	4.84n.s	26.39n.s	11.62**	1.07n.s	0.98*	2.67**	2.4**	257.31**	20.17**
Genotype (G)	16	17.58n.s	614.95*	3974.25**	468.61**	535.00**	45.99n.s	12.11*	126.37**	1549.86**	1657.32**
G × E	96	17.62**	351.03*	143.01**	66.73**	18.17**	31.93**	6.52**	49.86**	205.91**	185.03**
Error 2	224	0.19	4.27	22.69	1.66	0.69	0.54	0.75	1.01	49.69	6.59
CV		10.65	6.89	10.85	3.75	4.85	7.04	7.40	7.56	9.04	1.78

*, ** and ns: Significant (α= 5 %), highly significant (α= 1 %) and non-significant, respectively. CV: Coefficient of variation.

S5 Table. Mean comparison of genotype × environment interaction for grain yield of non-Iranian primary and combined primary tritipyrum lines, promising triticale lines, and bread wheat varieties under different environmental conditions.

Environments	Ka/b	La/b	La(4B/4D)/b	(Ma/b)×(Cr/b)-4	(Ka/b)×(Cr/b)-3	(Ka/b)×(Cr/b)-5	(Ka/b)×(Cr/b)-6	(St/b)×(Cr/b)-4	Triticale 4103	Triticale 4108	Triticale 4115	Triticale 4116	Triticale M45	Omid	Alvand	Baharebaft	Kavir
e_1	$2.32^{g'-v'}$	$1.58^{u'-y'}$	$3.65^{t-a'}$	$2.52^{d'-t'}$	$1.61^{t'-y'}$	4.25^{p-u}	$2.57^{d'-r'}$	1.80^{v-y}	4.20^{p-v}	5.20^{l-o}	3.76^{s-z}	7.04^{f-h}	4.29^{p-t}	9.87^{c}	$2.73^{b'-n'}$	$3.01^{y-i'}$	$2.84^{a'-j'}$
e_2	$1.86^{m'-y'}$	$1.78^{o'-y'}$	$1.89^{l'-y'}$	3.93^{r-x}	$2.16^{i'-v'}$	$2.77^{a'-m'}$	$3.19^{w-g'}$	$3.23^{w-g'}$	$2.45^{e'-u'}$	6.06^{j-k}	5.31^{k-n}	$2.73^{a'-n'}$	6.06^{j-k}	9.96^{c}	4.42^{o-t}	$2.91^{z-i'}$	6.49^{g-i}
e_3	7.38^{f}	10.69^{bc}	11.56^{a}	11.36^{a-b}	8.98^{d}	10.43^{c}	6.28^{h-j}	5.03^{l-p}	5.36^{k-n}	4.44^{o-t}	4.24^{o-t}	$3.40^{u-d'}$	7.85^{e-f}	$3.22^{w-g'}$	$1.84^{n'-y'}$	5.50^{j-m}	5.58^{j-l}
e_4	3.81^{s-y}	$1.92^{k'-y'}$	$2.07^{c'-o'}$	$1.03^{y'}$	$1.98^{j'-x'}$	$2.59^{d'-p'}$	$2.20^{i'-v'}$	$2.23^{i'-v'}$	$1.05^{y'}$	4.55^{n-s}	$2.87^{z-j'}$	$2.81^{a'-l'}$	4.42^{o-t}	$1.64^{s'-y'}$	$1.73^{p'-y'}$	$1.21^{w'-y'}$	$1.75^{p'-y'}$
e_5	$2.16^{i'-v'}$	$3.36^{t-b'}$	$1.74^{p'-y'}$	$2.91^{z-i'}$	4.8^{l-r}	5.64^{j-l}	$1.66^{q'-y'}$	$3.64^{t-a'}$	$2.41^{f'-v'}$	8.27^{d-e}	11.44^{ab}	7.83^{e-f}	$2.90^{z-j'}$	4.45^{o-t}	$3.59^{t-c'}$	10.21^{c}	$2.43^{e'-u'}$
e_6	$2.12^{i'-w'}$	$1.73^{p'-y'}$	$2.53^{d'-t'}$	$3.33^{v-f'}$	$2.46^{e'-u'}$	$3.02^{y-i'}$	$1.93^{k'-y'}$	$1.69^{q'-y'}$	7.09^{f-g}	4.98^{l-q}	8.67^{d}	4.03^{r-w}	8.67^{d}	4.72^{m-r}	$10.15i^{c}$	10.43^{c}	11.38^{ab}
e_7	$2.60^{d'-q'}$	$1.11^{x'y'}$	$2.87^{z-j'}$	$2.81^{a'-l'}$	$2.41^{f'-v'}$	$1.10^{x'y'}$	$2.63^{d'-p'}$	$2.75^{a'-n'}$	$1.51^{v'-y'}$	$3.35^{v-e'}$	$3.08^{x-h'}$	$1.88^{m'-y'}$	4.14^{q-v}	$1.83^{n'-y'}$	$2.55^{d'-s'}$	$2.57^{d'-r'}$	$1.18^{x'y'}$

Means in each column by similar letter(s) are not significantly different (α=5%), using Duncan's new multiple range test. e_1: Kerman (normal) and fourth crop year, e_2: Kerman (normal) and second crop year, e_3: Kerman (normal) and third crop year, e_4: Sirjan (normal) and fourth crop year, e_5: Neyriz (normal) and first crop year, e_6: Kerman (normal) and first crop year, and e_7: Sirjan (salinity) and fourth crop year.

S6 Table. Analysis of variance for grain yield of non-Iranian primary and combined primary tritipyrum lines, promising triticale lines, and bread wheat varieties in seven different environments using Eberhart and Russell's method.

Source of variation	Degrees of freedom	Sum of squares	Mean squares	F
Replication in Environment	14	1.69	0.28	4.67**
Genotype	16	93.94	5.87	1 n.s
Environment	6	243.19	40.53	6.66**
Genotype × Environment	96	563.61	5.87	8.00**
Environment (Linear)	1	243.19	243.19	-
Genotype × Environment (Linear)	16	66.54	4.16	0.71 n.s
Combined Deviation	85	497.07	5.85	97.50**
Ka/b	5	16.80	3.36	56.00**
La/b	5	27.25	5.45	90.83**
La(4B,4D)/b	5	40.75	8.15	135.83**
(Ma/b)(Cr/b)-4	5	23.7	4.74	79.00**
(Ka/b)(Cr/b)-3	5	16.2	3.24	54.00**
(Ka/b)(Cr/b)-5	5	17.00	3.40	56.67**
(Ka/b)(Cr/b)-6	5	9.50	1.90	31.67**
(St/b)(Cr/b)-4	5	6.15	1.23	20.50**
Triticale 4103	5	11.8	2.36	39.34**
Triticale 4108	5	13.7	2.74	45.67**
Triticale 4115	5	49.2	9.84	164.00**
Triticale 4116	5	29.35	5.87	97.83**
Triticale M45	5	15.9	3.18	53.00**
Omid	5	72.35	14.47	241.17**
Alvand	5	47.00	9.40	156.67**
Baharebaft	5	50.75	10.15	169.17**
Kavir	5	49.65	9.93	165.50**
Combined Error	224	14.53	0.06	-

** and ns: highly significant (α= 1 %) and non-significant, respectively.

S7 Table. Different stability parameters based on the regression method in new cereal, primary tritipyrum lines, promising triticale lines, and Iranian bread wheat varieties.

Genotypes	Mean yield ($t.ha^{-1}$)	Linear regression coefficient (b)	Intercept (a)	Sum of squares of the linear regression	Deviation from the linear regression	Linear coefficient of determination (R^2)
Ka/b	3.18	$0.66^{n.s}$	3.18	$6.21^{n.s}$	3.36^{**}	27.00
La/b	3.21	$1.71^{n.s}$	3.20	41.87^{*}	5.45^{**}	60.60
La(4B,4D)/b	3.85	$1.47^{n.s}$	3.85	$31.05^{n.s}$	8.15^{**}	43.20
(Ma/b)(Cr/b)-4	3.89	$1.76^{n.s}$	3.68	44.54^{*}	4.74^{**}	65.30
(Ka/b)(Cr/b)-3	3.48	$1.33^{n.s}$	3.49	25.47^{*}	3.42^{**}	61.10
(Ka/b)(Cr/b)-5	4.26	$1.66^{n.s}$	4.26	39.52^{*}	3.40^{**}	69.90
(Ka/b)(Cr/b)-6	3.92	$0.60^{n.s}$	2.92	$5.16^{n.s}$	1.90^{**}	35.20
(St/b)(Cr/b)-4	2.87	$0.45^{n.s}$	2.87	$2.92^{n.s}$	1.23^{**}	32.20
Triticale 4103	3.44	$1.1^{n.s}$	3.44	17.29^{*}	2.36^{**}	60.1
Triticale 4108	5.26	$0.26^{n.s}$	5.27	$0.99^{n.s}$	2.74^{**}	6.8
Triticale 4115	5.65	$0.96^{n.s}$	5.65	$13.06^{n.s}$	9.84^{**}	21.00
Triticale 4116	4.25	$0.37^{n.s}$	4.25	$2.00^{n.s}$	5.87^{**}	6.40
Triticale M45	5.48	$0.89^{n.s}$	5.48	$11.26^{n.s}$	3.18^{**}	41.40
Omid	5.10	$0.23^{n.s}$	5.10	$0.79^{n.s}$	14.47^{**}	1.10
Alvand	3.86	$0.56^{n.s}$	3.86	$4.55^{n.s}$	9.40^{**}	8.80
Baharebaft	5.12	$1.56^{n.s}$	5.12	$34.63^{n.s}$	10.15^{**}	40.60
Kavir	4.52	$1.41^{n.s}$	4.52	$28.42^{n.s}$	9.93^{**}	36.40

**: highly significant (α= 1 %).

S8 Table. Parameters of GE interactions for genotypes and environments in the AMMI3 model

**Genotypes**	ξ_1^1	ξ_2^2	ξ_3^3
Ka/b	-0.048	0.002	0.01
La/b	-0.057	0.004	-0.023
La(4B,4D)/b	-0.057	-0.016	0.005
(Ma/b)(Cr/b)-4	-0.053	-0.024	-0.003
(Ka/b)(Cr/b)-3	-0.051	0.019	-0.041
(Ka/b)(Cr/b)-5	-0.049	0.029	-0.017
(Ka/b)(Cr/b)-6	-0.029	0.004	0.076
(St/b)(Cr/b)-4	-0.021	0.041	0.011
4103	0.026	-0.06	0.013
4108	0.033	0.069	0.01
4115	0.045	0.034	-0.049
4116	0.027	0.074	0.007
M45	0.006	-0.084	0.029
Omid	0.027	0.024	0.078
Alvand	0.052	-0.042	-0.002
Bahare baft	0.039	0.003	-0.069
Kavir	0.034	-0.074	0.008
**Environments**	η_1	η_2	η_3
e_1	0.012	-0.04	0.072
e_2	0.026	0.010	-0.073
e_3	-0.057	0.017	0.016
e_4	-0.30	-0.014	-0.012
e_5	0.024	-0.064	0.057
e_6	0.045	0.056	0.026
e_7	-0.018	0.008	0.017

ζ1, ζ2, and ζ3 are the special genotype vectors for the first, second, and third component of the GE interaction, respectively.

η_1, η_2, and η_3 are the environmental vectors of the first, second, and third component of the GE interaction, respectively.

e_1: Kerman (normal) and fourth crop year, e_2: Kerman (normal) and second crop year, e_3: Kerman (normal) and third crop year, e_4: Sirjan (normal) and fourth crop year, e_5: Neyriz (normal) and first crop year, e_6: Kerman (normal) and first crop year, and e_7: Sirjan (salinity) and fourth crop year.

S9 Table. Eigen value and values of principal components of interaction effects for genotypes and environments in the AMMI3 model

Genotypes	Principal components of interaction effects		
	IPC1	IPC2	IPC3
Ka/b	-0.810	0.019	0.102
La/b	-0.950	0.039	-0.237
La(4B,4D)/b	-0.962	-0.173	0.053
(Ma/b)(Cr/b)-4	-0.885	-0.256	-0.028
(Ka/b)(Cr/b)-3	-0.863	0.200	-0.426
(Ka/b)(Cr/b)-5	-0.816	0.308	-0.179
(Ka/b)(Cr/b)-6	-0.492	0.041	0.496
(St/b)(Cr/b)-4	-0.349	0.443	0.110
Triticale 4103	0.430	-0637	0.133
Triticale 4108	0.551	0.734	0.103
Triticale 4115	0.751	0.363	-0.516
Triticale 4116	0.457	0.792	0.077
Triticale M45	0.099	-0.901	0.298
Omid	0.452	0.259	0.818
Alvand	0.870	-0.450	-0.025
Bahare baft	0.652	0.028	-0.723
Kavir	0.564	-0.787	0.082
Eigen value	281.2681	114.1301	108.9887
Cumulative% of components variance	49%	70%	89%
**Environments**	**IPC1**	**IPC2**	**IPC3**
e_1	0.193	-0.424	-0.754
e_2	0.434	0.110	-0.759
e_3	-0.956	0.186	0.162
e_4	-0.506	-0.147	-0.125
e_5	0.405	-0.684	0.596
e_6	0.747	0.600	0.267
e_7	-0.304	0.081	0.177
Eigen value	281.2681	114.1301	108.9887
Cumulative% of components variance	49%	70%	89%

e_1: Kerman (normal) and fourth crop year, e_2: Kerman (normal) and second crop year, e_3: Kerman (normal) and third crop year, e_4: Sirjan (normal) and fourth crop year, e_5: Neyriz (normal) and first crop year, e_6: Kerman (normal) and first crop year, and e_7: Sirjan (salinity) and fourth crop year.

S10 Table. AMMI3 model stability statistics for three hexaploid amphiploids including non-Iranian primary and combined primary tritipyrum lines, promising triticale lines, and bread wheat varieties in seven different environments.

Genotypes	$SIPC_3$	EV_3
Ka/b	0.931	0.0008
La/b	1.239	0.0013
La(4B,4D)/b	1.179	0.0012
(Ma/b)(Cr/b)-4	1.177	0.0011
(Ka/b)(Cr/b)-3	1.486	0.0015
(Ka/b)(Cr/b)-5	1.309	0.001
(Ka/b)(Cr/b)-6	1.323	0.0022
(St/b)(Cr/b)-4	0.905	0.0007
Triticale 4103	1.213	0.0015
Triticale 4108	1.395	0.002
Triticale 4115	1.629	0.0019
Triticale 4116	1.316	0.0021
Triticale M45	1.301	0.0026
Omid	1.524	0.0024
Alvand	1.342	0.00149
Baharebaft	1.406	0.0021
Kavir	1.444	0.0022

$SIPC_3$ and EV_3: stability parameters for AMMI3 model.

S11 Table. Correlation coefficients between grain yield and yield components for three hexaploid amphiploids including non-Iranian primary and combined primary tritipyrum lines, promising triticale lines, and bread wheat varieties.

Genotypes	Ka/b	La/b	La(4B,4D)/b	(Ma/b)(Cr/b)-4	(Ka/b)(Cr/b)-3	(Ka/b)(Cr/b)-5	(Ka/b)(Cr/b)-6	(St/b)(Cr/b)-4	
Correlation of grain yield with: Spike number	-0.49*	-0.55*	-0.30$^{n.s}$	-0.55*	-0.56**	-0.41$^{n.s}$	-0.47*	-0.65**	
Grain number per spike	0.06$^{n.s}$	-0.35$^{n.s}$	-0.06$^{n.s}$	-0.13$^{n.s}$	0.17$^{n.s}$	-0.21$^{n.s}$	-0.40$^{n.s}$	-0.32$^{n.s}$	
1000-grain weight	0.05$^{n.s}$	0.10$^{n.s}$	-0.18$^{n.s}$	-0.56**	-0.22$^{n.s}$	-0.31$^{n.s}$	-0.25$^{n.s}$	-0.48*	
Correlation of spike number with: Grain number per spike	-0.20$^{n.s}$	0.38$^{n.s}$	-0.12$^{n.s}$	-0.49*	-0.62**	-0.13$^{n.s}$	-0.06$^{n.s}$	0.27$^{n.s}$	
1000-grain weight	0.42$^{n.s}$	0.48*	0.70**	0.87**	0.67**	0.76**	0.57*	0.75**	
Correlation of grain number per spike with 1000-grain weight	0.17$^{n.s}$	0.12$^{n.s}$	0.27$^{n.s}$	-0.33$^{n.s}$	-0.49*	-0.21$^{n.s}$	-0.30$^{n.s}$	0.54*	

Genotypes	Triticale 4103	Triticale 4108	Triticale 4115	Triticale 4116	Triticale M45	Omid	Alvand	Baharebaft	Kavir
Correlation of grain yield with: Spike number	-0.51*	-0.36$^{n.s}$	-0.58**	-0.32$^{n.s}$	-0.48*	-0.18$^{n.s}$	0.05$^{n.s}$	-0.25$^{n.s}$	-0.65**
Grain number per spike	-0.26$^{n.s}$	-0.37$^{n.s}$	-0.27$^{n.s}$	0.08$^{n.s}$	-0.19$^{n.s}$	0.60**	0.59**	-0.03$^{n.s}$	-0.10$^{n.s}$
1000-grain weight	-0.27$^{n.s}$	-0.42$^{n.s}$	-0.62**	-0.05$^{n.s}$	-0.41$^{n.s}$	-0.10$^{n.s}$	0.09$^{n.s}$	-0.28$^{n.s}$	-0.60**
Correlation of spike number with: Grain number per spike	0.26$^{n.s}$	0.59**	0.56**	0.58**	0.87**	0.10$^{n.s}$	0.27$^{n.s}$	0.24$^{n.s}$	0.62**
1000-grain weight	0.69**	0.83**	0.72**	0.89**	0.67**	0.95**	0.67**	0.87**	0.93**
Correlation of grain number per spike with 1000-grain weight	0.69**	0.59**	0.21$^{n.s}$	0.60**	0.60**	0.16$^{n.s}$	0.56**	0.26$^{n.s}$	0.42$^{n.s}$

*, ** and ^{ns}: Significant (α= 0.05), highly significant (α= 0.01) and non-significant, respectively.

S12 Table. Path coefficients of grain yield and yield components for three hexaploid amphiploids including non-Iranian primary and combined primary tritipyrum lines, promising triticale lines, and bread wheat varieties in seven different environments.

**Genotypes**	a_1	a_2	a_3	a_4	a_5	a_6
Ka/b	-0.2010	0.4705	0.2632	-0.6631	-0.1330	0.3481
La/b	0.3810	0.5130	-0.0759	-0.7233	-0.1254	0.4609
La(4B,4D)/b	-0.1210	0.7470	0.3621	-0.4398	-0.1628	0.1746
(Ma/b)(Cr/b)-4	-0.4903	.09300	0.1259	-0.6743	-0.5065	-0.1369
(Ka/b)(Cr/b)-3	-0.6148	0.5907	-0.1268	-0.8811	-0.2502	0.2521
(Ka/b)(Cr/b)-5	-01315	0.7441	-0.1134	-0.4098	-0.2761	-0.0532
(Ka/b)(Cr/b)-6	-0.0627	0.5563	-0.2641	-0.4116	-0.4656	-0.1492
(St/b)(Cr/b)-4	0.2686	0.6542	0.3589	-0.7336	-0.2199	0.1903
Triticale 4103	0.2579	0.5448	0.5529	-0.8280	-0.4986	0.6463
Triticale 4108	0.5874	0.7290	0.1631	0.0146	-0.1809	-0.3281
Triticale 4115	0.5582	0.8845	0.2873	-0.2307	-0.0466	-0.4407
Triticale 4116	0.5776	0.8176	0.1224	-1.3671	0.2719	0.9986
Triticale M45	0.8689	0.6227	0.0559	-1.1652	0.9416	-0.1923
Omid	0.1014	0.9471	0.0633	-0.6139	0.5985	0.3936
Alvand	0.2658	0.5563	0.4075	-0.0597	0.7752	-0.3002
Baharebaft	0.2369	0.8574	0.0555	-0.0471	0.0468	-0.2475
Kavir	0.6160	1.0764	-0.2392	-1.7449	0.6547	0.7464

a_1-a_6 are path coefficients of spike number with grain number per spike, spike number with 1000-grain weight, grain number per spike with 1000-grain weight, spike number with grain yield, grain number per spike with grain yield, and 1000-grain weight with grain yield, respectively.

S13 Table. Genotypic components of GE interaction for three hexaploid amphiploids including non-Iranian primary and combined primary tritipyrum lines, promising triticale lines, and bread wheat varieties.

**Genotypes**	$\mu_{i.}$	σ_{wi}	V_1	V_2	V_3
Ka/b	3.18	1.869	-0.918	-0.077	0.566
La/b	3.207	3.225	-1.767	-0.477	1.297
La(4B,4D)/b	3.848	3.298	-0.980	0.327	0.353
(Ma/b)(Cr/b)-4	3.984	3.202	-1.745	-1.463	-0.211
(Ka/b)(Cr/b)-3	3.484	2.510	-1.403	-0.560	0.467
(Ka/b)(Cr/b)-5	4.258	2.926	-1.211	-0.784	-0.099
(Ka/b)(Cr/b)-6	2.923	1.496	-0.700	-0.636	-0.248
(St/b)(Cr/b)-4	2.865	1.220	-0.793	-0.178	0.131
Triticale 4103	3.439	2.100	-1.075	-0.288	0.668
Triticale 4108	5.263	1.562	-0.565	-0.297	-0.281
Triticale 4115	5.651	3.081	-1.775	0.203	-0.878
Triticale 4116	4.247	2.256	-0.729	0.726	1.011
Triticale M45	5.475	2.038	-0970	0.940	-0.289
Omid	5.099	3.378	-0.601	2.095	0.392
Alvand	3.857	2.793	-0.148	1.757	-0.533
Baharebaft	5.122	3.641	-0914	1.165	-0.441
Kavir	4.522	3.437	2.227	1.289	0.818

$\mu_{i.}$ and σ_{wi}: mean and standard deviation of grain yield of ith genotype, respectively. V_1-V_3: genotypic components of stability.

S14 Table. Environmental components of GE interaction for different environments in Kerman, Sirjan and Neyriz.

Environments	r_1	r_2	r_3
e_1	0.231	0.443	0.675
e_2	0.412	0.732	-0.266
e_3	-1.832	-1.453	2.539
e_4	1.154	-1.170	-0.251
e_5	-0.893	0.487	-1.066
e_6	-0.111	2.065	0.786
e_7	1.038	-1.105	0.845

r_1-r_3 are environmental components influencing on the spike number, grain number per spike and 1000-grain weight, respectively. e_1: Kerman (normal) and fourth crop year, e_2: Kerman (normal) and second crop year, e_3: Kerman (normal) and third crop year, e_4: Sirjan (normal) and fourth crop year, e_5: Neyriz (normal) and first crop year, e_6: Kerman (normal) and first crop year, and e_7: Sirjan (salinity) and fourth crop year.

S15 Table. Spearman's rank correlations coefficients between different stability statistics three hexaploid amphiploids including non-Iranian primary and combined primary tritipyrum lines, promising triticale lines, and bread wheat varieties.

	CV_i	S^2_i	μ	b_i	S^2_{di}	R^2_i	W^2_i	σ^2_i	IPC_1	IPC_2	IPC_3	$SIPC_3$	EV_3
CV_i	1.00												
S^2_i	0.99**	1.00											
μ	-1.00**	-0.99**	1.00										
b_i	1.00**	0.99**	-1.00**	1.00									
S^2_{di}	1.00**	0.99**	-1.00**	1.00**	1.00								
R^2_i	-1.00**	-0.99**	1.00**	-1.00**	-1.00**	1.00							
W^2_i	1.00**	0.99**	-1.00**	1.00**	1.00**	-1.00**	1.00						
σ^2_i	1.00**	0.99**	-1.00**	1.00**	1.00**	-1.00**	1.00**	1.00					
IPC_1	1.00**	0.99**	-1.00**	1.00**	1.00**	-1.00**	1.00**	1.00**	1.00				
IPC_2	1.00**	0.99**	-1.00**	1.00**	1.00**	-1.00**	1.00**	1.00**	1.00**	1.00			
IPC_3	1.00**	0.99**	-1.00**	1.00**	1.00**	-1.00**	1.00**	1.00**	1.00**	1.00**	1.00		
$SIPC_3$	1.00**	0.99**	-1.00**	1.00**	1.00**	-1.00**	1.00**	1.00**	1.00**	1.00**	1.00**	1.00	
EV_3	0.99**	0.99**	-0.99**	0.99**	0.99**	-0.99**	0.99**	0.99**	0.99**	0.99**	0.99**	0.99**	1.00

**: Highly significant (α= 0.01), CV_i: Coefficient of variation, S^2_i: Environmental variance, μ : Mean yield, b_i: Regression coefficient, S^2_{di}: Deviation from regression line, R^2_i: Coefficient of determination, W^2_i: Wrick ecovalance, σ^2_i: Shukla stability variance, IPC_1, IPC_2 and IPC_3: First, second and third principal components in AMMI model, respectively, $SIPC_3$ and EV_3: AMMI model stability statistics.
